# Function through bio-inspired, synthesis-informed design: step-economical syntheses of designed kinase inhibitors†
†Dedicated to Max Malacria, a friend and scholar whose science and creative contributions to step-economical synthesis have inspired us all and moved the field closer to the ideal.
‡
‡Electronic supplementary information (ESI) available: Synthetic procedures and spectral data. See DOI: 10.1039/c4qo00228h



**DOI:** 10.1039/c4qo00228h

**Published:** 2014-10-06

**Authors:** Paul A. Wender, Alison D. Axtman, Jennifer E. Golden, Jung-Min Kee, Lauren E. Sirois, Ryan V. Quiroz, Matthew C. Stevens

**Affiliations:** a Department of Chemistry and Department of Chemical and Systems Biology , Stanford University , Stanford , CA 94305 , USA . Email: wenderp@stanford.edu

## Abstract

The human kinome comprises over 500 protein kinases. When mutated or over-expressed, many play critical roles in abnormal cellular functions associated with cancer, cardiovascular disease and neurological disorders. Here we report a step-economical approach to designed kinase inhibitors inspired by the potent, but non-selective, natural product staurosporine, and synthetically enabled by a novel, complexity-increasing, serialized [5 + 2]/[4 + 2] cycloaddition strategy. This function-oriented synthesis approach rapidly affords tunable scaffolds, and produced a low nanomolar inhibitor of protein kinase C.

## Abstract 2

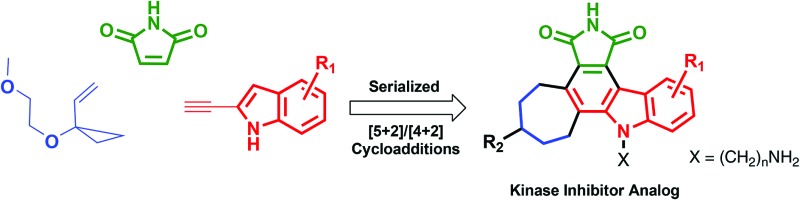
We describe here step-economical, function-oriented strategies towards the syntheses of potent kinase inhibitors inspired by the natural product staurosporine.

## Introduction

**Fig. 1:**
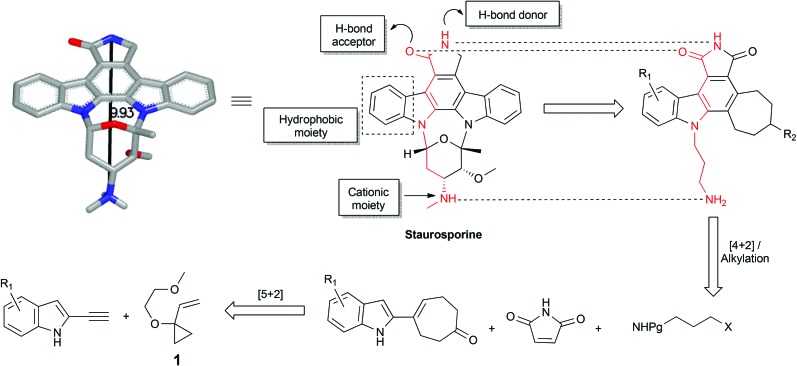
Design of staurosporine analogs based on [5 + 2]/[4 + 2] serialized cycloadditions.

Protein kinases catalyse the transfer of a γ-phosphate from ATP to select serine, threonine or tyrosine residues in a range of client proteins, thereby regulating cellular pathways central to proliferation, metabolism, apoptosis, transport, differentiation, and cell–cell signaling.^1–4^ Over-expression and/or mutation of these proteins disrupt homeostatic functions of consequence in cancer, cardiovascular, neuronal, inflammatory, and metabolic diseases.^5,6^ Not surprisingly, compounds that control kinase activity by activation (regulatory domain) or inhibition (regulatory and ATP binding site) have emerged as preeminent clinical candidates or drugs for numerous therapeutic indications. For example, prostratin, a potent protein kinase C (PKC) modulator, is a preclinical lead for the eradication of HIV/AIDS.^7^ Bryostatin, also a PKC modulator, is in clinical trials for the treatment of Alzheimer's disease.^8^ Kinase inhibitors have also fuelled research interest, leading to the preclinical and clinical advancement of many ATP-binding site inhibitors.^9–11^ Due to the highly conserved nature of the ATP-binding site, engineering selectivity is one of the fundamental challenges associated with the discovery or design of new ATP-competitive kinase inhibitors.^12^ While early views on whether selectivity at the ATP-binding site could be achieved were understandably mixed, subsequent studies have shown that structural tuning of certain inhibitor scaffolds can indeed lead to selective inhibition. Lilly scientists, for example, reported the clinical candidates ruboxistaurin^13^ for diabetic retinopathy, and the cancer therapeutic enzastaurin,^14^ each of which exhibited high target selectivity (60-fold and 26-fold selectivity for PKCβ over PKCα, respectively). These and related studies^15,16^ provide compelling motivation for the search for new, more effective and accessible scaffolds that feature readily amendable functionalities for selectivity optimization. Prompted by our ongoing interest in PKC regulators,^7b,8a,17,18^ we initiated a program several years ago in which computer-guided, synthesis-informed design would be employed to create and make new ATP-competitive inhibitors. Rather than screening libraries for lead structures, we sought to identify, through X-ray crystal structure analyses and computer-based design, the activity determining features of potent but non-selective natural leads and incorporate those features into designed but simpler structures that could be readily accessed and tuned through synthesis, in essence creating function through bio-inspired, computer-guided, and synthesis-informed design.^19^ Staurosporine (Fig. 1), a promiscuous inhibitor isolated in 1977 ^20^ that affects over 253 kinases with a *K*_d_ less than 3 μM ^21^ (and is therefore too toxic to be used as a drug), served as a potent starting point for implementing this function-oriented synthesis strategy, especially given the difficulty of synthesis of the natural product itself.^22,23^ Staurosporine's inhibitory function is achieved in part through complementary hydrogen-bonding and electrostatic interactions involving its lactam subunit and cationic amine with its kinase host.^24^ These two groups are situated on orthogonally related indolocarbazole and carbohydrate subunits and spaced by approximately 9.93 Å (PKCθ, PDB code ; 1XJD, Fig. 1). From a design perspective, we expected that these key pharmacophoric features could be emulated by an unsymmetrical cycloheptenylcarbazole that would separate a tethered amine on average by a controllable 8–11 Å distance from the maleimide nitrogen. An attractive aspect of this scaffold is that it is designed to be convergently assembled from four readily available fragments in a step-, time- and atom-economical fashion^25,26^ using back-to-back complexity increasing cycloadditions. Specifically, a metal-catalysed [5 + 2] cycloaddition^27–31^ of commercially available vinylcyclopropane (VCP) **1** with an alkynyl indole would chemospecifically produce a cycloadduct whose diene subunit is poised to engage maleimide or similar dienophiles in a subsequent Diels–Alder [4 + 2] cycloaddition (Fig. 1). These back-to-back one-flask cycloadditions would thus allow for a rapid build-up of molecular complexity and structural features that could be easily modified to affect biological activity. Incorporation of a tunable alkyl-tethered amine would then complete the pharmacophore features while functionality in the seven-membered ring could be used for diversification (*e.g.* reductive amination) to control selective association with the ATP binding site. The successful execution of this design and synthesis strategy is detailed herein.

## Results and discussion

**Scheme 1:**
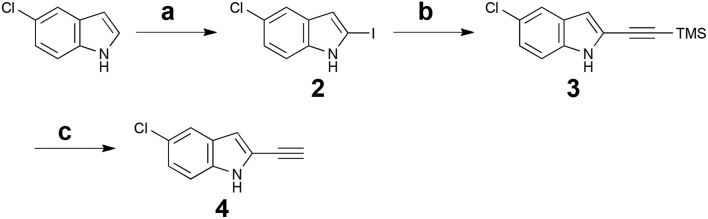
Synthesis of 5-chloro-2-ethynylindole **4**. Reagents and conditions: (a) i. *n*-BuLi, THF, –78 °C, then CO_2_ (g); ii. *t*-BuLi, –78 °C, 1 h, then 1,2-diiodoethane, –78 °C, 20 min, 73%. (b) 5 mol% Pd(PPh_3_)_2_Cl_2_, 10 mol% CuI, (trimethylsilyl)acetylene, Et_3_N, 40 °C, 30 min, 98%. (c) NaOH (aq.), i-PrOH, RT, 15 min, 92%.

Cycloadditions allow for the convergent assembly of simple starting materials into complex products, producing two new bonds and generally up to four new stereocenters. Serialized cycloadditions significantly amplify this complexity increase. We previously reported the use of serialized [5 + 2]/[4 + 2] cycloadditions in which the ynophilicity of a Rh-catalysed [5 + 2] cycloaddition was exploited to convert an enyne to a diene which was then trapped in a Diels–Alder or metal catalysed [4 + 2] cycloaddition to produce a polycyclic product.^32^ If now the enyne were derived from an indole scaffold, coupled with maleimide as a dienophile, the resulting polycycloaddition products would incorporate the activity-determining features of staurosporine but based on a simpler, more accessible scaffold (Fig. 1). Based on this function-oriented synthesis (FOS) analysis,^19^ this step-economical strategy would allow one to achieve scaffold diversification through variations in any of the four building blocks of the multi-component design. To explore this concept, ethynylindole **4** was prepared in three steps from commercial 5-chloroindole (Scheme 1). The chloroindole was chosen based on previously reported SAR studies with bisindolylmaleimide compounds,^33^ as well as the fact that the chlorine could serve as a functional handle for late-stage diversification *via* cross-coupling strategies. Installation of an iodine at the 2-position of the chloroindole by Bergman's method,^34^ subsequent Sonogashira coupling^35^ with (trimethylsilyl)acetylene, and deprotection provided step-economical access to desired alkynyl indole **4**.

**Scheme 2:**
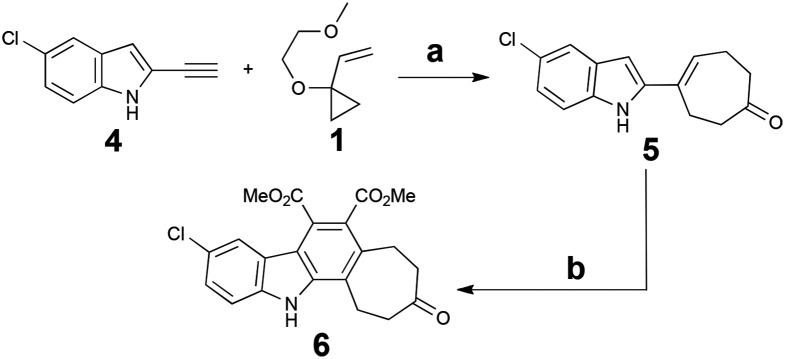
Synthesis of the key core scaffold **6**. Reagents and conditions: (a) 5 mol% [Rh(CO)_2_Cl]_2_, DCE, RT, 18 h, then H^+^, 61%. (b) Dimethyl acetylenedicarboxylate, PhMe, reflux, 72 h; then DDQ, RT, 30 min, 57%.

Notwithstanding potential interference from the indole NH, the Rh-catalysed [5 + 2] cycloaddition of alkyne **4** with commercially available VCP **1** proceeded at room temperature and provided upon hydrolytic workup cycloadduct **5** in good yield. However, attempts to subsequently capture the diene produced *in situ* with maleimide proved complex. As a result, recourse to a two-step procedure was explored based on dimethyl acetylenedicarboxylate (DMAD). This proved effective, yielding the key core structure **6** upon oxidative workup (Scheme 2).

**Scheme 3:**
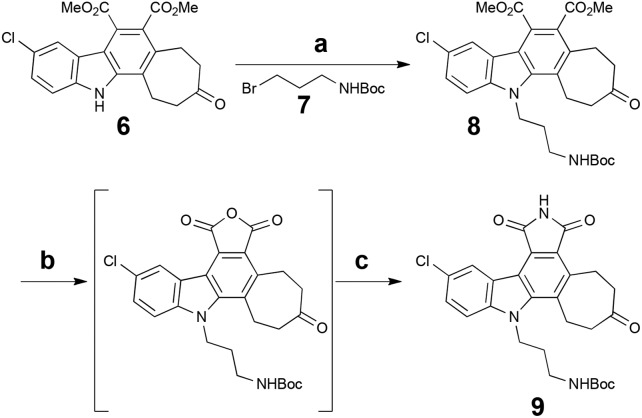
Completed synthesis of pentacyclic common intermediate **9**. Reagents and conditions: (a) Cs_2_CO_3_, **7**, DMF, 75 °C, 14 h, 77%. (b) KOH (aq.), EtOH, 70 °C, 22 h; then citric acid, RT, 10 min. (c) HMDS, MeOH, CH_3_CN, reflux, 12 h, 57% (two steps).

To complete the synthesis and arrive at a common intermediate for diversification, the indole nitrogen of **6** was alkylated with side chain **7**, followed by hydrolysis of the two esters and subsequent acidification to yield the cyclic anhydride. Treatment with hexamethyldisilazane^36^ (HMDS) then afforded the final pentacyclic core, **9**, in 8 steps overall from commercial materials. Importantly, **9** possesses three differentiated functionalities (aryl chloride, cycloheptanone, alkyl tether), allowing for regional diversification of this scaffold to modulate biological properties (Scheme 3).

**Scheme 4:**
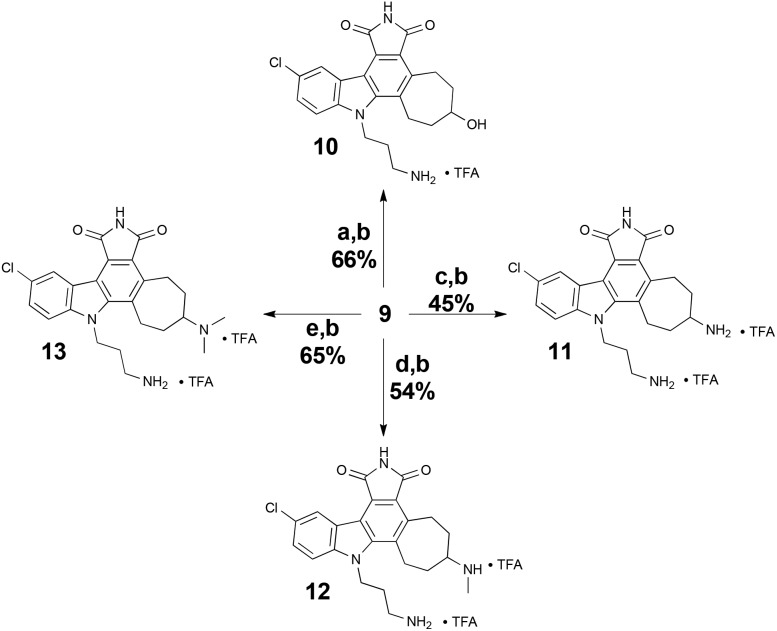
Ketone diversification of **9** for analog generation. Reagents and conditions: (a) NaBH_4_, MeOH–CH_2_Cl_2_ (1 : 1), 0 °C, 10 min. (b) TFA, i-Pr_3_SiH, CH_2_Cl_2_, RT. (c) NH_4_OAc, Et_3_N, NaCNBH_3_, MeOH–CH_2_Cl_2_ (1 : 1), RT, 4 h. (d) MeNH_3_Cl, Et_3_N, NaCNBH_3_, MeOH–CH_2_Cl_2_ (1 : 2), RT, 3 h. (e) Me_2_NH_2_Cl, Et_3_N, NaCNBH_3_, MgSO_4_, MeOH–CH_2_Cl_2_ (1 : 2), RT, 48 h.

Our first diversification study was directed at the ketone functionality of **9**, as computer modelling suggested it would be positioned in a region of the ATP-binding site largely unexplored in studies related to the staurosporine scaffold. As such, ketone **9** was subjected to reducing conditions with sodium borohydride and various reductive amination procedures for biological assessment (Scheme 4).

Our initial analogs were tested for inhibition of PKC^37,38^ (rat brain mixture) due to its clinical significance and our on-going and complementary work on PKC activators.^7b,16,26^ The results for these inhibition assays are summarized in Table 1.

**Table 1:** PKC inhibition results for select analogs

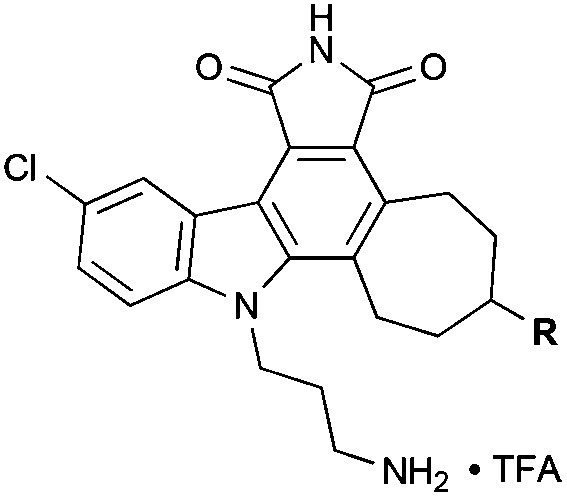		
Compound	R	IC_50_ (μM)
**10**	–OH	2.6
**11**	–NH_3_^+^	12.5
**12**	–NH_2_Me^+^	14.1
**13**	–NHMe_2_^+^	2.9

**Scheme 5:**
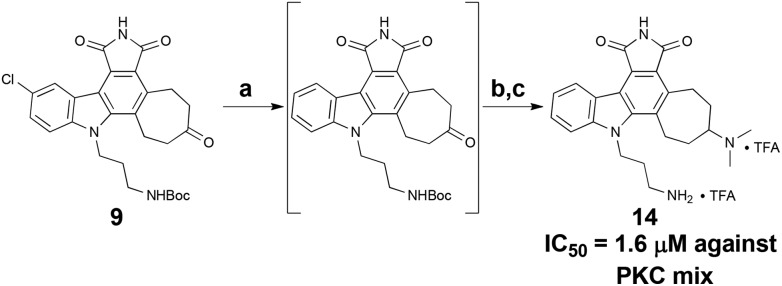
De-chlorination to provide analog **14**. Reagents and conditions: (a) Pd(OAc)_2_, KF, PMHS, H_2_O–THF (2 : 5), RT, 1 h. (b) Me_2_NH_2_Cl, Et_3_N, NaCNBH_3_, RT, 48 h. (c) TFA, i-Pr_3_SiH, DCM, RT, 30 min, 43% (3 steps).

While less potent than staurosporine, these initial analogs showed that qualifying activity could be readily realized through this conceptual approach, thus prompting its further investigation and refinement. Interestingly, the cycloheptyl alcohol and dimethylamino analogs, **10** and **13**, were the most potent. The methoxy analog of compound **10** was synthesized, but its potency was greatly reduced compared to the alcohol variant (data not shown), demonstrating overall the need for a hydrogen-bond donor in this region. Finally, to investigate whether the chlorine was necessary for potency, a palladium-mediated de-chlorination^39^ was carried out. Interestingly, for PKC we found that this compound (**14**) had improved potency (1.6 μM) over the corresponding chlorinated derivative **13** (Scheme 5).

**Fig. 2:**
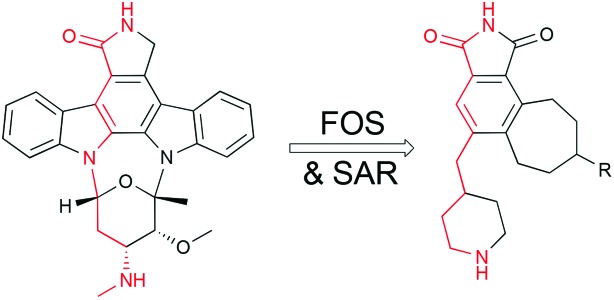
Design of a simplified staurosporine analog.

Because deletion of the indole chlorine was not detrimental, we next sought to determine whether the indole subunit itself was necessary, as its deletion would simplify the target and further shorten the synthesis. The proposed analogs would retain the pharmacophoric features on a tricyclic (7-6-5) framework still accessible through the [5 + 2]/[4 + 2] cycloaddition sequence, with the pendant amine incorporated as a conformationally constrained piperidine moiety to limit entropic penalties for kinase binding (Fig. 2).

**Scheme 6:**
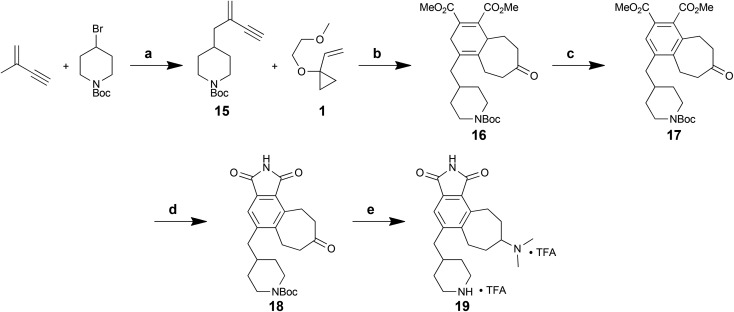
Synthesis of a new des-indole analog **19**. Reagents and conditions: (a) *n*-BuLi, KO*t*Bu, LiBr, THF, –78 °C to –20 °C to 0 °C, 1.5 h, 66%. (b) [(C_10_H_8_)Rh(COD)]SbF_6_, DCE, RT, 45 min; then DMAD, RT, 15 min; then H^+^, 75%. (c) DDQ, PhMe, 80 °C, 6.5 h, 84%. (d) 1 N KOH (aq.), EtOH, 80 °C, 2–4 h; then Ac_2_O, 50 °C, 18 h; then HMDS–MeOH (2 : 1), DMF, 80 °C, 3 h, 41% (3 steps). (e) NaBH_3_CN, Me_2_NH_2_Cl, Et_3_N, mol. sieves, MeOH–DCM (1 : 2), RT, 48 h; then TFA, i-Pr_3_SiH, DCM, RT, 30 min, 67%.

The synthetic route to these new analogs is shown below (Scheme 6). Briefly, piperidinyl enyne **15**, readily prepared in one step from commercial starting materials,^40^ was subjected to a Rh-catalysed [5 + 2] cycloaddition with VCP **1**. In this case, the diene formed *in situ* was successfully trapped with DMAD in a [4 + 2] cycloaddition to afford overall polycycle **16** in a one-flask procedure. Oxidation of the central cyclohexadiene core with DDQ proceeded smoothly to give **17**. Next, a three-step protocol (ester hydrolysis, formation of the maleic anhydride, then opening and re-closing with HMDS) was used to convert the diester to maleimide **18** in 41% overall yield over 3 steps. Finally, reductive amination of the cycloheptyl ketone and deprotection of the amine afforded final analog **19** in 8 steps overall from commercially available starting materials.

**Fig. 3:**
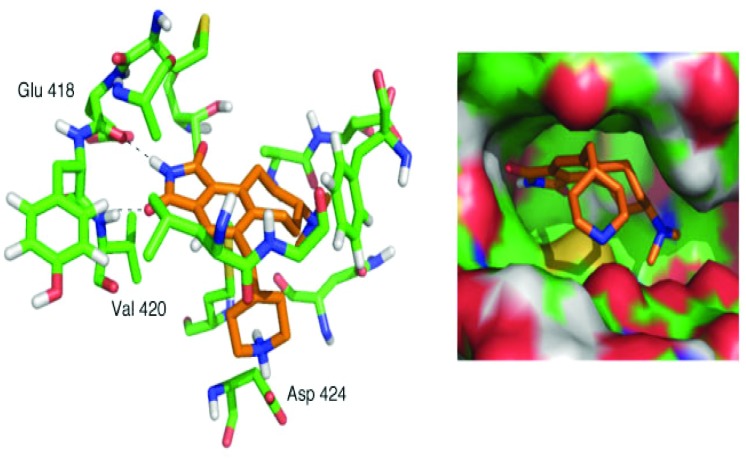
Computer docking of energy-minimized analog **19** into the active site of PKCα.

Simplified analog **19** was tested in the PKC inhibition assay using the rat brain mixture of PKC isoforms. Gratifyingly, **19** exhibited a potent IC_50_ value of 13 nM. This value closely approaches that of staurosporine at 2.7 nM,^41^ yet the scaffold itself is much more readily accessible and tunable. Computational docking^42^ of the energy-minimized conformer of **19** in the computer-simulated ATP-binding pocket of PKCα (Fig. 3) illustrates the conserved hydrogen bonds between the maleimide and hinge region residues and the shape complementarity of the ligand with the pocket. Overall, this FOS strategy, enabled through design and through the use of back-to-back, one-flask powerful cycloaddition processes, provides rapid access to active kinase inhibitor scaffolds that can be readily modified and that potently inhibit enzymatic activity.

## Conclusion

In summary, through an iterative process involving function- and synthesis-informed design, we have synthesized staurosporine-inspired kinase inhibitors that can be readily accessed and tuned employing a novel, complexity-increasing [5 + 2]/[4 + 2] cycloaddition strategy. Our initial indole analogs inhibited PKC in the low micromolar range. Further simplification of this series, involving deletion of the indole moiety, gave rise to a second analog series with low nanomolar activity now approaching that of the lead natural product, staurosporine. This initial proof-of-concept study successfully demonstrated that significant potency can be achieved through design based on a novel multicomponent cycloaddition series. The flexibility of these syntheses has enabled current studies aimed at achieving selective inhibition through scaffold modulation. More generally, this study illustrates how the confluence of bio-inspired and computer-aided design, informed by new reactions ([5 + 2]) or novel synthetic strategies (serialized [5 + 2]/[4 + 2] cycloadditions),^31^ can be used to achieve practical kinase inhibitory function. Further studies on these novel kinase inhibitors are underway as are more general studies directed at achieving step-economically accessed function through synthesis-informed design.
