# Supplementary material for: Function through bio-inspired, synthesis-informed design: step-economical syntheses of designed kinase inhibitors
Source: Org Chem Front. 2014 Oct 6;1(10):1166–71. doi: 10.1039/c4qo00228h (PMC4304288; doi:10.1039/c4qo00228h)
Supplement: Supplementary file 1 [file QO-001-C4QO00228H-s001.pdf]

Electronic Supplementary Material (ESI) for Organic Chemistry Frontiers.
This journal is © the Partner Organisations 2014

Supporting Information for

Function through Bio-Inspired, Synthesis-Informed Design: Step-Economical Syntheses of Designed Kinase Inhibitors

Wender, P. A.; Axtman, A. D.; Golden, J. E.; Kee, J. -M.; Sirois, L. E.; Quiroz, R. V.; Stevens, M. C.

Department of Chemistry and Department of Chemical and Systems Biology, Stanford University, Stanford, CA 94305, United States of America

wenderp@stanford.edu

Table of Contents

General Methods……………………………………………………………**2-3**

Synthetic Procedures and Compound Data………………………………**4-23**

PKC Inhibition Assay……………………………………………………….**24-25**

NMR Spectra…………………………………………………………………**26-42**

General Methods

Unless otherwise noted, air- and moisture-sensitive reactions were carried out in oven-dried (>110 ºC) glassware capped with rubber septa under a positive pressure of dry nitrogen or argon from a manifold or balloon. Likewise, air- and moisture-sensitive reagents, solvents, and solutions were transferred via syringe or stainless steel cannula under a dry inert atmosphere. Stirring was achieved using oven-dried (>110 ºC) Teflon®-coated magnetic stir bars cooled under a stream of dry nitrogen or argon.

Reactions were run at room temperature (rt, 20-25 °C) unless otherwise noted in the experimental procedure. Elevated temperatures were maintained through the use of a silicone oil bath, which was equilibrated under constant current through a wire-heating element. For reactions below room temperature, the term "-78 °C" refers to a bath of acetone and dry ice, "-40 °C" refers to a slurry of acetonitrile and dry ice, "-20 ºC" refers to a slurry of NaCl salt and ice, and "0 °C" refers to an ice-water bath. "Concentration" of solvent refers to removal of solvent using a Büchi rotary evaporator equipped with a portable vacuum pump. Removal of residual solvents was accomplished by evacuation of the vessel using a high vacuum line maintained at 0.1-1.0 torr.

Reagents and Solvents

Unless otherwise specified, all commercial reagents and solvents were purchased from Aldrich Chemical Company, TCI America, or Acros Organics and were used without further purification with the following exceptions. $[(C_{10}H_8)Rh(COD)]SbF_6$ catalyst was synthesized according to literature procedures.[1] Vinylcyclopropane **1** was purchased from Aldrich. Tetrahydrofuran, diethyl ether, dichloromethane, and toluene were purified via passage through an activated alumina column (*Solv-Tek*, Inc.). Dichloroethane, pyridine, diisopropylethylamine, triethylamine, and 2,6-lutidine were distilled from calcium hydride under a positive pressure of nitrogen.

Chromatographic and Spectroscopic Methods

Thin layer chromatography (TLC) was performed using 250 µm glass-backed, silica gel 60 F254 coated plates from Merck. The plates were visualized by ultraviolet

light (254 nm) and treatment with acidic p-anisaldehyde stain or potassium permanganate stain followed by gentle heating on a hot plate. Proton and carbon NMR spectra were measured on a Varian Inova-300 (^{1}H at 300 MHz, ^{13}C at 75 MHz), Varian Mercury-400 (^{1}H at 400 MHz, ^{13}C at 100 MHz), Varian Inova-500 (^{1}H at 500 MHz, ^{13}C at 125 MHz), or a Varian Inova-600 (^{1}H at 600 MHz, ^{13}C at 150 MHz). Chemical shifts are reported in parts per million (ppm) downfield from tetramethylsilane and referenced to the solvent peak (for $CDCl_3$: ^{1}H NMR 7.26 ppm, ^{13}C NMR 77.16 ppm; *d*-DMSO: ^{1}H NMR 2.50 ppm, ^{13}C NMR 39.52 ppm; *d*-acetone: ^{1}H NMR 2.05 ppm, ^{13}C NMR 29.84 ppm; CD_3OD: ^{1}H NMR 3.31 ppm, ^{13}C NMR 49.00 ppm). Abbreviations are used in the reported description of NMR data as follows: chemical shift (δ, ppm), multiplicity (s = singlet, d = doublet, dd = doublet of doublets, dt = doublet of triplets, dq = doublet of quartets, ddd = doublet of doublets of doublets, ddt = doublet of doublets of triplets, dtd = doublet of triplets of doublets, t = triplet, td = triplet of doublets, q = quartet, quin = quintet, m = multiplet, br = broad, and coupling constant (J, Hz). Data for ^{13}C are reported in terms of chemical shift and quantity of carbons (in the case of overlapping signals). High-resolution mass spectroscopy (HRMS) was recorded at the Vincent Coates foundation mass spectrometry laboratory at Stanford University, California. The samples were analyzed by LC/ESI-MS on a Waters Acquity UPLC and Thermo Fisher Exactive mass spectrometer scanning m/z 100-1000. The LC conditions were 90% Solvent A (0.1% formic acid in water) ramped to 100% Solvent B (0.1% formic acid in acetonitrile) in 3 minutes, and held for 1 minute. The column was a 2.1x50mm Zorbax 300 SB C18 Rapid Resolution 1.8u and the flow rate was 0.2mL/min. Infrared (IR) spectra were recorded on a Perkin-Elmer™ 1600 series FTIR spectrometer. IR data is reported as frequencies in wavenumbers (cm^{-1}). Samples were prepared as thin films on a NaCl salt plate.

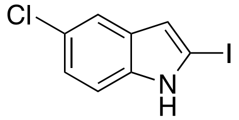

5-chloro-2-iodo-1*H*-indole (2) Following Bergman's method[2], in a round-bottom flask, 5-chloroindole (5.30 g, 34.9 mmol) was dissolved in dry THF (70 mL) and cooled to –78 ºC under argon atmosphere. *n*-butyllithium (32.4 mL, 1.13 M solution in hexanes) was added via a syringe dropwise over 10 min at –78 °C. After the resulting white suspension was stirred for 30 min at –78 °C, CO_2 (g) was bubbled through the reaction mixture for 15 min, and the obtained clear solution was stirred at –78 °C for an additional 10 min. The solution was carefully warmed up to rt over 1 h while maintaining good ventilation to remove the CO_2 gas. The solvent was removed using a rotary evaporator to obtain a pale yellow foamy solid. After the solid residue was dissolved in dry THF (70 mL) at rt under an argon atmosphere, the clear solution was cooled down to –78 °C, and *tert*-butyllithium (27.5 mL, 1.33 M solution in pentane) was added via a syringe dropwise over 25 min. The resulting yellow-orange solution was stirred for 1 h at –78 °C. A solution of 1,2-diiodoethane (9.74 g, 34.9 mmol) in dry THF (15 mL) was added dropwise over 30 sec. On addition, the apricot reaction mixture turned colorless and turbid. After the mixture was stirred for 20 min at –78 °C, saturated aqueous NH_4Cl (10 mL) was added, and it was allowed to warm up to rt. Water (20 mL) and diethyl ether (50 mL) were added and the dark brown organic phase was collected, and the aqueous phase was extracted twice with diethyl ether (50 mL). The combined organic phases was washed with saturated aqueous $Na_2S_2O_3$ and brine, dried over anhydrous $MgSO_4$, and concentrated *in vacuo* to afford a yellow-brown syrup. The crude residue was purified by flash column chromatography (5% Et_2O/petroleum ether) to give the product as a white solid (5.83 g, 73 %).

TLC $\boldsymbol{R_f}$ = 0.52 (diethyl ether : petroleum ether = 3 : 7), one orange spot stained with p-anisaldehyde (visible under UV lamp).

mp (crystallized from pentane) = 92-93 °C (decomp.).

^{1}H NMR (300 MHz, $CDCl_3$) δ = 8.08 (br, s, 1H), 7.49 (d, 1H, J = 2.0 Hz), 7.21 (d, 1H, J = 8.8 Hz), 7.07 (dd, 1H, J = 2.0 Hz, 8.6 Hz), 6.64 (t, 1H, J = 1.0 Hz) ppm.

IR (KBr) ν = 3399, 3388, 2959, 2155, 1449, 1398, 1344, 1249, 983, 841 cm^{-1}.

^{13}**C NMR** (75 MHz, $CDCl_3$) δ = 137.2, 130.6, 126.2, 122.7, 118.7, 112.4, 111.1, 76.5 ppm.

HRMS (*m/z*) : Calculated : 276.9155 (for [M^+] C_8H_5ClIN); found, 276.9159.

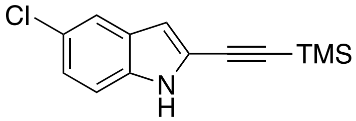

5-chloro-2-((trimethylsilyl)ethynyl)-1*H*-indole (3) In a 3-neck round-bottom flask equipped with a magnetic stir-bar, CuI (94.0 mg, 0.486 mmol), 5-chloro-2-iodoindole **2** (1.35 g, 4.86 mmol), and $Pd(PPh_3)_2Cl_2$ (174 mg, 0.243 mmol) were dissolved in triethylamine (40 mL) at rt under nitrogen atmosphere. The yellow solution was warmed up to 40 °C and trimethylsilylacetylene (1.05 mL, 7.29 mmol) was added via a syringe over 4 min. Within 10 min, a yellow-gray precipitate was formed. After 30 min, the reaction mixture turned black, and TLC indicated the reaction was complete. The reaction mixture was cooled to rt and poured into diethyl ether (100 mL). Aqueous 3 N HCl (70 mL) was carefully added and the colorless organic phase was removed. The aqueous phase was extracted with diethyl ether (4 x 50 mL) and the combined organic phases were washed with saturated aqueous NH_4Cl (100 mL) and brine (100 mL), dried over $MgSO_4$, and concentrated *in vacuo*. The resultant brown syrup was purified by flash column chromatography (10% diethyl ether/pentane) to give the product as a pale orange crystalline solid (1.179 g, 98% yield).

TLC R_f = 0.80 (30% diethyl ether/pentane), one purple spot stained with p-anisaldehyde (visible under UV lamp).

mp (crystallized from pentane) = 81-82 ºC.

^{1}**H NMR** (300 MHz, $CDCl_3$) δ = 8.18 (brs, 1H), 7.53 (d, 1H, J = 0.7 Hz), 7.18 (d, 1H, J = 8.8 Hz), 7.15 (dd, 1H, J = 1.8 Hz, 8.7 Hz), 6.88 (d, 1H, J = 1.5 Hz), 0.266 (s, 9H) ppm.

^{13}**C NMR** (75 MHz, $CDCl_3$) δ = 134.3, 128.6, 126.3, 124.1, 120.29, 120.14, 111.8, 108.8, 99.4, 96.5, -0.1 ppm.

IR (KBr) ν = 3428, 2960, 2157, 1465, 1397, 1334, 1250, 1063, 859 cm^{-1}.

HRMS (*m/z*) : Calculated: 247.0584 (for [M^+] $C_{13}H_{14}ClNSi$); found: 247.0579.

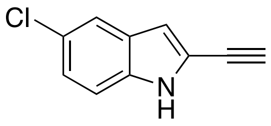

5-chloro-2-ethynyl-1H-indole (4) In a round-bottom flask equipped with a magnetic stir bar, indole **3** (2.09 g, 8.42 mmol) was dissolved in isopropanol (42 mL) under a nitrogen atmosphere. Aqueous 1N NaOH (42 mL) was added via a syringe over 10 min at rt. The reaction mixture turned turbid immediately. After 15 min, water (200 mL) was added and the organic phase was collected. After the aqueous phase was extracted with diethyl ether (100 mL), the combined organic phase was washed with saturated aqueous NH_4Cl (50 mL) and brine (50 mL), dried over $MgSO_4$, and concentrated *in vacuo*. The brown residue was purified by flash column chromatography (15% diethyl ether/pentane) to give the product as pale orange amorphous solid (1.36 g, 92% yield).

TLC $\boldsymbol{R_f}$ = 0.40 (30% Et_2O/pentane), one purple spot stained with p-anisaldehyde (visible under UV lamp).

^{1}H NMR (300 MHz, $CDCl_3$) δ = 8.21 (brs, 1H), 7.55 (d, 1H, J = 1.7 Hz), 7.24 (d, 1H, J = 9.0 Hz), 7.19 (dd, 1H, J = 1.8 Hz, 8.7 Hz), 6.75 (d, 1H, J = 1.8 Hz), 3.33 (s, 1H) ppm.

^{13}C NMR (75 MHz, CDCl3) δ = 134.2, 128.4, 126.3, 124.2, 120.3, 119.0, 112.0, 109.2, 81.6, 75.8 ppm.

IR (KBr) ν = 3396, 3299, 1396, 1333, 868, 797, 677, 614 cm^{-1}.

HRMS (*m/z*): Calculated: 175.0189 (for [M^+] $C_{10}H_6ClN$); found: 175.0185.

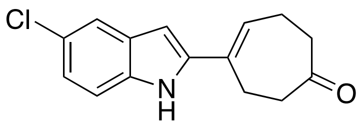

4-(5-chloro-1*H*-indol-2-yl)cyclohept-4-en-1-one (5) In a round-bottom flask equipped with a magnetic stir bar, ethynylindole **4** (532 mg, 3.03 mmol) and $[Rh(CO)_2Cl]_2$ (62.3 mg, 0.155 mmol) were dissolved in dry 1,2-dichloroethane (DCE, 10 mL) at rt under a nitrogen atmosphere. Immediately, vinylcyclopropane **1** (627 mg, 4.23 mmol) was added via a syringe in one portion, and the flask wall was rinsed using additional DCE (5 mL). The mixture was stirred at rt for 18 h under a nitrogen atmosphere until TLC indicated complete consumption of **4**. Then 1% ethanolic HCl (0.1 mL) and water (0.1 mL) were added and the reaction mixture was stirred for 1 h and was passed through a plug of silica gel using ethyl acetate (20 mL). The dark brown crude mixture was concentrated *in vacuo* and purified by flash column chromatography (30% EtOAc/ pentane) to give the product as an orange amorphous solid (492 mg, 61% yield).

TLC R_f = 0.65 (Et_2O), one green spot stained with p-anisaldehyde (visible under UV lamp).

^{1}H NMR (300 MHz, d_6-acetone) δ = 10.48 (brs, 1H), 7.48 (d, 1H, J = 1.7 Hz), 7.31 (d, 1H, J = 8.6 Hz), 7.04 (dd, 1H, J = 2.0 Hz, 8.6 Hz), 6.51 (m, 2H), 2.83-2.87 (m, 2H), 2.65-2.70 (m, 2H), 2.54-2.63 (m, 4H) ppm.

^{13}C NMR (75 MHz, d_6-acetone) δ = 211.21, 142.32, 136.63, 134.30, 131.00, 125.34, 122.69, 120.19, 112.98, 100.00, 99.91, 42.81, 42.67, 26.29, 24.52 ppm.

IR (KBr) ν = 3352, 1925, 1698, 1447, 1309, 1060, 916, 787 cm^{-1}.

HRMS (*m/z*) : Calculated : 259.0764 (for [M^+] $C_{15}H_{14}ClNO$); found: 259.0763.

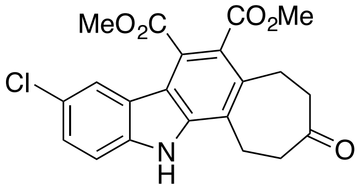

Dimethyl 9-chloro-3-oxo-1,2,3,4,5,12-hexahydrocyclohepta[*a*]carbazole-6,7-dicarboxylate (6) In a Teflon-capped pressure vessel, diene **5** (200 mg, 0.770 mmol) and dimethyl acetylenedicarboxylate (DMAD, 0.120 mL, 1.26 mmol) were dissolved in dry toluene (6.7 mL) under a nitrogen atmosphere. The vessel was sealed and heated to 115 ºC for 48 h. An additional portion of DMAD (50 μl) was added and the mixture

was heated at 115 ºC for additional 15 h. Another portion of DMAD (20 μl) was added and the mixture was heated to 145 ºC for 9 h until TLC indicated complete consumption of the starting material **5**. After the reaction mixture was cooled down to rt, 2,3-dichloro-5,6-dicyanoquinone (DDQ, 74 mg, 0.32 mmol) was added portionwise over 30 min until the intermediate diene was completely converted to the product (monitored by TLC). The crude mixture was filtered through a pad of silica gel using ethyl acetate (30 mL) as the eluent. The filtrate was washed with saturated aqueous $NaHCO_3$ (10 mL), water (10 mL) and brine (2 × 10 mL) respectively, and was dried over $MgSO_4$ and concentrated *in vacuo* to give a dark brown oil. The crude residue was purified by flash column chromatography (15% EtOAc/dichloromethane) to give the desired product as a yellow powder (110.4 mg, 42% yield).

TLC R_f = 0.26 (Et_2O), one purple spot stained with p-anisaldehyde (visible under UV lamp).

^{1}H NMR (500 MHz, d_6-DMSO) δ = 11.99 (s, 1H), 8.00 (d, 1H, J = 1.9 Hz), 7.58 (dd, 1H, J = 6.6 Hz, 0.5 Hz), 7.50 (dd, 1H, J = 8.7 Hz, 2.2 Hz), 3.96 (s, 3H), 3.84 (s, 3H), 3.33-3.37 (m, 2H), 3.08-3.10 (m, 2H), 2.65-2.67 (m, 2H), 2.57-2.60 (m, 2H) ppm.

^{13}C NMR (125 MHz, d_6-DMSO) δ = 209.5, 168.8, 167.9 140.0, 139.5, 136.5, 127.3, 126.8, 124.2, 123.6, 123.5, 122.0, 121.7, 116.6, 113.1, 52.9, 52.6, 43.5, 42.5, 25.3, 22.5 ppm.

IR (KBr) ν = 3350, 2951, 1720, 1601, 1434, 1285, 1230, 1134, 1117, 1068, 803, 730 cm^{-1}.

HRMS (*m/z*) : Calculated: 399.0874 (for [M+] $C_{21}H_{18}ClNO_5$); found: 399.0871.

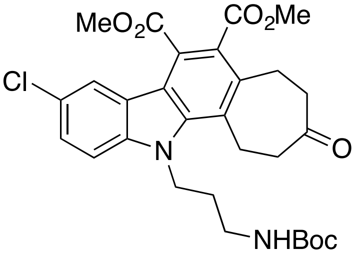

Dimethyl 12-(3-((*tert*-butoxycarbonyl)amino)propyl)-9-chloro-3-oxo-1,2,3,4,5,12-hexahydrocyclohepta[*a*]carbazole-6,7-dicarboxylate (8) To a round-bottom flask equipped with a reflux condenser, carbazole **6** (132 mg, 0.329 mmol), bromide **7** (234

mg, 0.987 mmol), and Cs_2CO_3 (750 mg, 2.30 mmol) were added under nitrogen atmosphere. Dry DMF (7.8 mL) was added to the mixture, and the resultant deep red suspension was stirred at 75 ºC for 14 h until TLC indicated the complete consumption of the starting carbazole. The reaction mixture was cooled to rt and filtered through filter paper using ethyl acetate (20 mL) to wash the filtered solid. The filtrate was poured into saturated aqueous NH_4Cl (20 mL) and the organic phase was collected. After the aqueous phase was extracted with ethyl acetate (3 × 10 mL), the combined organic phases were washed with water and brine, dried over $MgSO_4$, and concentrated *in vacuo* to give a brown syrup. The crude residue was purified by flash column chromatography (20% EtOAc/dichloromethane) to give the desired product as a very pale yellow oil (140.7 mg, 77% yield).

TLC R_f = 0.55 (20% EtOAc/pentane), one purple spot stained with p-anisaldehyde (visible under UV lamp).

^{1}H NMR (300 MHz, $CDCl_3$) δ = 7.96 (d, 1H, J = 1.9 Hz), 7.39 (dd, 1H, J = 2.0 Hz, 8.8Hz), 7.25 (d, 1H, J = 8.8 Hz), 4.74 (s, 1H), 4.31 (t, 2H, J = 7.9 Hz), 4.04 (s, 3H), 3.91 (s, 3H), 3.36-3.40 (m, 2H), 3.12-3.21 (m, 4H), 2.73 (m, 2H), 1.99 (q, 1H, J = 7.6 Hz), 1.44 (s, 9H) ppm.

^{13}C NMR (75 MHz, $CDCl_3$) δ = 209.21, 169.09, 168.63, 156.15, 140.82, 139.44, 138.19, 127.17, 126.39, 125.54, 124.86, 123.97, 121.78, 121.57, 118.65, 110.16, 79.61, 65.42, 53.01, 52.70, 44.02, 43.54, 37.92, 30.45, 28.40, 26.07, 22.71 ppm.

IR (KBr) ν = 3390, 2952, 2252, 1714, 1574, 1450, 1248, 1168, 994, 913, 800, 732 cm^{-1}.

HRMS (*m/z*) : Calculated: 556.1976 (for [M^+] $C_{29}H_{33}ClN_2O_7$); found: 556.1960.

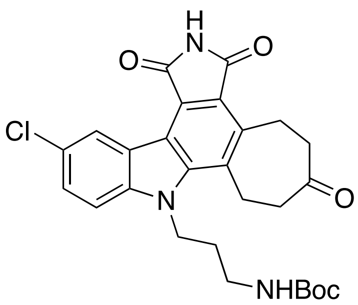

***tert*-butyl (3-(12-chloro-1,3,6-trioxo-1,2,3,4,5,6,7,8-octahydro-9*H*-cyclohepta[*a*]pyrrolo[3,4-*c*]carbazol-9-yl)propyl)carbamate (9)** In a round-bottom

flask equipped with a reflux condenser, diester **8** (174.5 mg, 0.313 mmol) was suspended in ethanol (6.0 mL). 1 N aqueous KOH (2.0 mL) was added in one portion via a syringe at rt and the suspension was heated to 70 ºC. The mixture turned clear within 5 min. After 12 h, KOH (70 mg) in water (1.0 mL) was added and the reaction mixture was refluxed for an additional 10 h until TLC indicated complete consumption of the starting material. The reaction mixture was cooled down to rt and diluted with water (10 mL). Dichloromethane (10 mL), and 20% citric acid (10 mL) were added and stirred for 10 min. The organic phase was collected and the aqueous phase was extracted with dichloromethane (4 × 10 mL). The combined organic phases were washed with water (10 mL) and brine (2 ×10 mL), and concentrated *in vacuo* to afford a yellow oil. Absolute ethanol (5 mL) was added and evaporated to yield ~200 mg of a yellow powder.

In a round-bottom flask equipped with a reflux condenser, the crude product was dissolved in dry acetonitrile (30 mL) and heated to a gentle reflux at 80 ºC. A mixture of HMDS (3.00 mL, 14.2 mmol) and methanol (0.30 mL, 7.4 mmol) was added in one portion, and the resultant suspension was refluxed for 12 h. Methanol (6 mL) was added and the turbid mixture was refluxed for 15 min. The solvent was evaporated and the resultant yellow-brown oil was purified by flash column chromatography (20 % ethyl acetate in dichloromethane) to give the desired product as a bright yellow powder (90.2 mg, 57% for two steps).

TLC R_f = 0.78 (EtOAc), one purple spot stained with p-anisaldehyde (visible under UV lamp).

^{1}H NMR (500MHz, d_6-DMSO) δ = 11.13 (s, 1H), 8.79 (d, 1H, J = 1.9 Hz), 7.69 (d, 1H, J = 8.8 Hz), 7.51 (dd, 1H, J = 2.2 Hz, 8.7 Hz), 7.13 (t, 1H, J = 5.6 Hz), 4.39 (t, J = 7.7 Hz, 2H), 3.68 (t, J = 6.0 Hz, 2H), 3.39 (m, 2H), 3.11 (d, 2H, J = 5.9 Hz), 2.74 (m, 2H), 2.56 (m, 2H), 1.96 (t, 2H, J = 7.2 Hz), 1.40 (s, 9H) ppm.

^{13}C NMR (125 MHz, d_6-DMSO) δ = 209.2, 170.8, 169.4, 155.7, 141.7, 141.6, 139.4, 130.3, 127.9, 125.0, 124.6, 123.7, 120.9, 120.8, 117.8, 111.6, 77.8, 43.9, 43.2, 43.1, 37.3, 30.1, 28.3, 21.9, 21.1 ppm.

IR (KBr) ν = 3401, 3215, 2976, 1701, 1458, 1400, 1337, 1278, 1164, 1087, 729 cm^{-1}.

HRMS (*m/z*): Calculated: 509.1717 (for [M^+] $C_{27}H_{28}ClN_3O_5$); found: 509.1715.

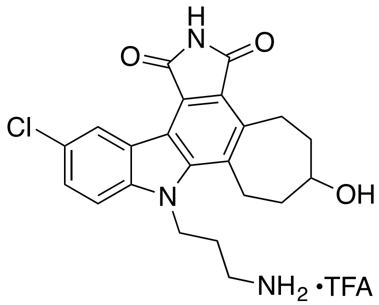

***tert*-butyl (3-(12-chloro-6-hydroxy-1,3-dioxo-1,2,3,4,5,6,7,8-octahydro-9*H*-cyclohepta[*a*]pyrrolo[3,4-*c*]carbazol-9-yl)propyl)carbamate (10)** In a round-bottom flask equipped with a magnetic stir bar, ketone **9** (12.1 mg, 0.024 mmol) was dissolved in dry methanol (1.5 mL) and dichloromethane (1.5 mL) at rt. After the solution was chilled to 0 ºC, sodium borohydride (2.6 mg, 0.069 mmol) was added in one portion and the reaction mixture was stirred for 10 min until TLC indicated complete consumption of the starting ketone. The mixture was diluted with EtOAc (5 mL) and quenched with aqueous NH_4Cl solution (5 mL). The organic phase was collected, and the aqueous phase was extracted with EtOAc (2 × 5 mL). Combined organic phases were washed with brine (5 mL) and concentrated *in vacuo* to afford a yellow amorphous solid. This crude residue was used directly in the next step.

In a round bottom flask equipped with a magnetic stir bar, the crude residue was suspended in dry dichloromethane (1.0 mL) and triisopropylsilane (33 µL, 0.16 mmol) was added in one portion. Trifluoroacetic acid (TFA, 300 µL, 4.2 mmol) was added dropwise via a syringe over 30 sec at rt. On addition of TFA, the suspension turned into a clear solution. After 30 min, the reaction mixture was diluted with dry dichloromethane (5 mL) and concentrated under reduced pressure to afford a yellow-brown residue. The crude product was purified by reverse-phase HPLC (Alltech Alltima C18 10u (250 mm × ID 22 mm, 5→95% acetonitrile/water gradient, flow rate = 20 mL/min) to give the desired produce (9.7 mg, 66% yield) as a bright yellow powder after lyophilization.

^{1}H NMR (600MHz, d_6-DMSO) δ = 11.27 (s, 1H), 9.12 (d, 1H, J = 1.8 Hz), 7.92 (d, 1H, J = 9.0 Hz), 7.87 (brs, 3H), 7.23 (dd, 1H, J = 1.7 Hz, 9.1 Hz), 6,63 (s, 1H), 4.92 (s, 1H),

4.71 (t, 2H, J = 7.8 Hz), 4.02 (brs, 2H), 3.62 (dd, 1H, J = 10.2 Hz, 15 Hz), 3.11 (dd, 1H, J = 10.2 Hz, 14.4 Hz), 2.99 (brs, 2H), 2.17 (m, 3H), 200 (brs, 1H), 1.70 (brs, 1H), 1.58 (brs, 1H) ppm.

^{13}C NMR (125 MHz, d_6-DMSO) δ = 171.0, 169.6, 142.0, 141.9, 141.7, 133.2, 127.8, 124.6, 123.9, 121.3, 121.1, 118.7, 117.9, 111.9, 81.2, 80.2, 43.1, 36.5, 36.4, 35.2, 34.9, 27.8, 21.8, 20.8 ppm.

IR (KBr) ν = 3435, 2927, 1691, 1457, 1204, 1138, 1089, 802 cm^{-1}.

HRMS (*m/z*): Calculated: 412.1423 (for [M^+] $C_{22}H_{23}ClN_3O_3$); found: 412.1425.

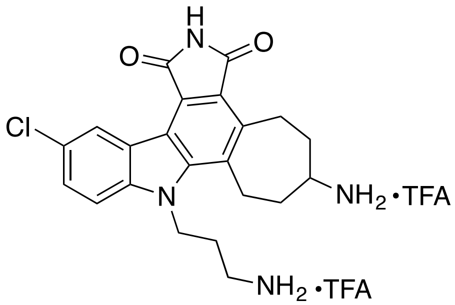

6-amino-9-(3-aminopropyl)-12-chloro-4,5,6,7,8,9-hexahydro-1*H*-cyclohepta[*a*]pyrrolo[3,4-*c*]carbazole-1,3(2*H*)-dione (11) In a round-bottom flask equipped with a magnetic stir bar, ketone **9** (11.0 mg, 0.021 mmol) and ammonium acetate (45 mg, 0.57 mmol) were dissolved in dry methanol (1.0 mL) and dichloromethane (1.0 mL) at room temperature. To the resultant yellow solution, triethylamine (9.0 µL, 0.064 mmol) was added and the mixture was stirred for 10 minutes at room temperature. Sodium cyanoborohydride (2.0 mg, 0.030 mmol) was added in one portion, and the slightly turbid mixture was stirred under a nitrogen atmosphere for 4 h. Additional sodium cyanoborohydride (0.9 mg, 0.014 mmol) was added and the reaction mixture was stirred for 1 h until TLC indicated complete consumption of ketone **9**. The mixture was diluted with dichloromethane (5 mL) and quenched with aqueous sodium bicarbonate solution (5 mL) and 3N aqueous NaOH solution (0.5 mL). The organic phase was removed, and the aqueous phase was extracted with dichloromethane (3 × 5 mL). Combined organic phases were washed with brine (5 mL) concentrated *in vacuo* to afford a yellow amorphous solid.

In a round bottom flask, the crude residue was suspended in dry dichloromethane (1.0 mL) and triisopropylsilane (43 μL, 0.21 mmol) was added in one portion. Trifluoroacetic acid (TFA, 160 μL, 2.1 mmol) was added dropwise via a syringe over 30 seconds at rt. On addition of TFA, the suspension turned into a clear solution. After 30 minutes, the reaction mixture was diluted dry dichloromethane (5 mL) and concentrated under reduced pressure to afford a yellow-brown residue. The crude product was purified by reverse-phase HPLC (Alltech Alltima C18 10u (250 mm * ID 22 mm, 5→95% acetonitrile/water gradient, flow rate = 20 mL/min) to give the desired product (6.0 mg, 45% yield) as a bright yellow powder after lyophillization.

^{1}H NMR (500MHz, d_6-DMSO) δ = 11.24 (s, 1H), 9.02 (d, 1H, J = 2.4 Hz), 7.98 (brs, 3H), 7.83 (d, 1H, J = 9.0 Hz), 7.63 (dd, 1H, J = 2.4 Hz, 8.6 Hz), 4.61 (m 2H),4.30 (dd, 1H, J = 7.8 Hz, 14.4 Hz), 3.57 (dd, 1H, J = 8.4 Hz, 15.0 Hz), 350 (brs, 1H), 3.07 (dd, 1H, J = 10.6 Hz, 15.6 Hz), 2.97 (t, 1H, J = 12.9 Hz), 2.90 (brs, 1H), 2.84 (brs, 1H), 2.36 (t, 1H, J = 8.7 Hz), 2.23 (brs, 1H), 2.04 (m, 2H), 1.43 (dd, 1H, J = 11.6 Hz, 23 Hz), 1.34 (dd, 1H, J = 11.9 Hz, 23 Hz) ppm.

^{13}C NMR (125 MHz, d_6-DMSO) δ = 171.7, 168.7, 158.7, 154.2, 142.6, 142.2, 141.0, 132.3, 131.9, 128.7, 125.8, 124.7, 122.0, 121.7, 118.9, 116.8, 112.7, 73.9, 53.0, 46.4, 37.1, 28.5, 24.5, 23.6, 22.6 ppm.

IR (KBr) ν = 3438, 3178, 1688, 1531, 1458, 1405, 1340, 1281, 1254, 1204, 1183, 1133, 1087, 1027, 1001, 900, 839, 799, 767, 722, 691, 646, 615 cm^{-1}.

HRMS (*m/z*): Calculated: 411.1582 (for [M+H$^+$] $C_{22}H_{24}ClN_4O_2$); found 411.1582.

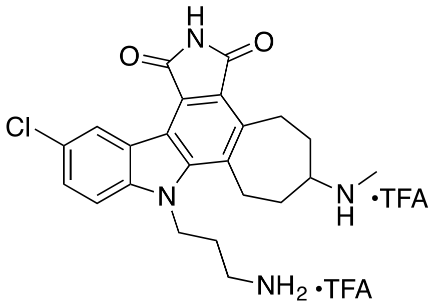

9-(3-aminopropyl)-12-chloro-6-(methylamino)-4,5,6,7,8,9-hexahydro-1*H*-cyclohepta[*a*]pyrrolo[3,4-*c*]carbazole-1,3(2*H*)-dione (12) In a round-bottom flask

equipped with a magnetic stir bar, ketone **9** (12.8 mg, 0.025 mmol) and methylamine hydrochloride (52 mg, 0.77 mmol) were dissolved in dry methanol (0.8 mL) and dichloromethane (1.6 mL) at rt. To the resultant yellow solution, triethylamine (10.5 µL, 0.075 mmol) was added and the mixture was stirred for 10 min at rt. Sodium cyanoborohydride (2.0 mg, 0.030 mmol) was added in one portion, and the slightly turbid mixture was stirred under a nitrogen atmosphere for 3 h until TLC indicated complete consumption of ketone **9**. The mixture was diluted with dichloromethane (5 mL) and quenched with aqueous ammonium chloride solution (5 mL). The organic phase was collected, and the aqueous phase was extracted with dichloromethane (2 × 5 mL). Combined organic phases were washed with brine (5 mL) concentrated *in vacuo* to afford a yellow amorphous solid.

In a round bottom flask, the crude residue was suspended in dry dichloromethane (1.2 mL) and triisopropylsilane (52 µL, 0.25 mmol) was added in one portion. Trifluoroacetic acid (TFA, 195 µL, 2.5 mmol) was added dropwise via a syringe over 30 sec at rt. On addition of TFA, the suspension turned into a clear solution. After 30 min, the reaction mixture was diluted dry dichloromethane (5 mL) and concentrated *in vacuo* to afford a yellow-brown residue. The crude product was purified by reverse-phase HPLC (Alltech Alltima C18 10u (250 mm × ID 22 mm, 5→95% acetonitrile/water gradient, flow rate = 20 mL/min) to give the desired product (8.9 mg, 54% yield) as a bright yellow powder after lyophillization.

^{1}H NMR (500MHz, *d_6*-DMSO) δ = 11.25 (s, 1H), 9.01 (s, 1H), 8.77 (brs, 2H), 7.93 (brs, 3H), 7.86 (d, 1H, J = 8.8 Hz), 7.65 (d, 1H, J = 8.8 Hz), 4.64 (m, 2H), 4.33 (dd, 1H, J = 8.4 Hz, 13.8 Hz), 3.62 (dd, 1H, J = 9.0 Hz, 15.6 Hz), 3.45 (m, 1H), 3.09 (dd, 1H, J = 10.4 Hz, 15.6 Hz), 2.90 – 3.00 (m, 3H), 2.59 (s, 3H), 2.35 (m, 1H) 2.09 (m, 2H), 1.50 (q, 1H, J = 12.0 Hz), 1.40 (q, 1H, J = 12.0 Hz) ppm.

^{13}C NMR (125 MHz, *d_6*-DMSO) δ = 171.63, 170.19, 159.00, 158.76, 158.52, 142.52, 142.22, 140.84, 132.06, 128.69, 125.73, 125.49, 124.62, 121.97, 121.66, 118.81, 112.62, 60.18, 43.75, 37.06, 30.08, 29.16, 28.97, 28.48, 23.49, 22.51 ppm.

IR (KBr) ν = 3432, 1686, 1458, 1203, 1132, 800, 722 cm^{-1}.

HRMS (*m/z*): Calculated: 425.1739 (for [M+H^{+}] $C_{23}H_{26}ClN_4O_2$); found: 425.1725.

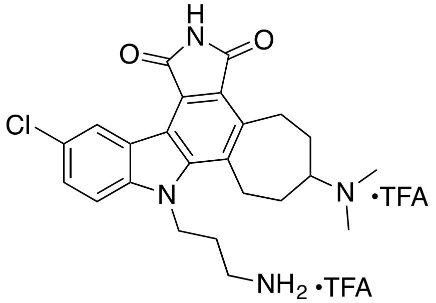

9-(3-aminopropyl)-12-chloro-6-(dimethylamino)-4,5,6,7,8,9-hexahydro-1*H*-cyclohepta[*a*]pyrrolo[3,4-*c*]carbazole-1,3(2*H*)-dione (13) In a round-bottom flask equipped with a magnetic stir bar, ketone **9** (14.2 mg, 0.0278 mmol) and Me_2NH_2Cl (70.2 mg, 0.835 mmol) were dissolved in methanol (0.9 mL) and dichloromethane (1.8 mL) at rt under a nitrogen atmosphere. Triethylamine (11.6 μl, 0.083 mmol) was added in one portion at rt. After 30 min, $NaCNBH_3$ (2.2 mg, 0.033 mmol) and anhydrous $MgSO_4$ (25 mg) were added in one portion respectively. The reaction mixture was stirred for 48 h at rt until TLC indicated complete consumption of ketone **9**. Dichloromethane (5 mL) and saturated aqueous NH_4Cl (5 mL) were added, and the organic phase was collected. 3N aqueous NaOH (3 mL) was added to the aqueous phase that was subsequently extracted with dichloromethane (2 × 5 mL). The combined organic phases were washed with brine (10 mL) and water (10 mL), dried over $MgSO_4$, filtered, and concentrated *in vacuo* to give a yellow powder.

The crude amine was suspended in dichloromethane (1.4 mL), and *i*Pr_3SiH (57 μl, 0.28 mmol) was in one portion. Subsequently, trifluoroacetic acid (215 μl, 2.78 mmol) was added dropwise over 30 sec to afford a bright yellow solution. After 30 min, dichloromethane (5 mL) was added and the reaction mixture was concentrated in vacuo to give a yellow solid. The crude product was purified by reverse-phase HPLC (Alltech Alltima C18 10u (250 mm × ID 22 mm, 5→95% acetonitrile/water gradient, flow rate = 20 mL/min) to give the desired product (12.4 mg, 65% yield) as a bright yellow powder after lyophillization.

^{1}H NMR (500MHz, d_6-DMSO) δ = 11.27 (brs, 1H), 10.01 (brs, 1H), 9.01 (d, 1H, J = 2.2 Hz), 7.94 (brs, 3H), 7.85 (d, 1H, J = 9.0 Hz), 7.65 (dd, 1H, J = 2.2 Hz, 8.8 Hz), 4.63 (m, 2H), 4.48 (dd, 1H, J = 7.8 Hz, 14.4 Hz), 3.68 (dd, 2H, J = 8.4 Hz, 15.0 Hz), 3.01

(dd, 1H, J = 11.4 Hz, 15.0 Hz), 2.92 (m, 2H), 2.84 (t, 1H, J = 13.2 Hz), 2.70 (s, 6H), 2.48 (m, 1H), 2.36 (m, 1H), 2.11 (t, 2H, J = 7.5 Hz), 1.60 (q, 1H, J = 12.0 Hz), 1.49 (q, J = 12.0 Hz) ppm.

^{13}C NMR (125 MHz, *d*$_6$-DMSO) δ = 171.67, 170.20, 159.27, 159.02, 158.77, 158.53, 142.52, 142.25, 140.87, 132.14, 128.73, 125.70, 125.50, 124.63, 121.97, 121.63, 119.14, 118.84, 116.75, 112.58, 67.30, 43.76, 39.51, 37.05, 28.51, 26.78, 26.66, 23.67, 22.68 ppm.

IR (KBr) ν = 3432, 2927, 1686, 1458, 1205, 1130, 802, 723 cm^{-1}.

HRMS (*m/z*): Calculated: 439.1895 (for [M+H$^+$] $C_{24}H_{28}ClN_4O_2$); found 439.1882.

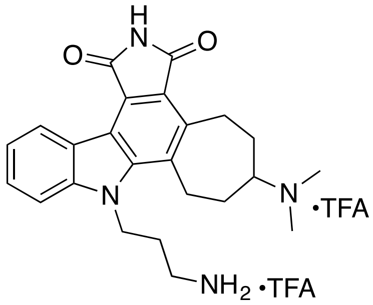

9-(3-aminopropyl)-6-(dimethylamino)-4,5,6,7,8,9-hexahydro-1*H*-cyclohepta[*a*]pyrrolo[3,4-*c*]carbazole-1,3(2*H*)-dione (15) In a conical flask equipped with a magnetic stir bar, ketone **9** (11.0 mg, 0.022 mmol) was dissolved in THF (0.3 mL) and water (0.12 mL) to give a bright yellow solution. KF (3.5 mg, 0.060 mmol) and $Pd(OAc)_2$ (2.4 mg, 0.011 mmol) were added in one portion respectively to the solution at rt under a nitrogen atmosphere. Polymethylhydrosiloxane (PMHS, 8 μL) was added via a microsyringe in one portion, and the mixture was stirred at rt for 1 h until TLC indicated complete consumption of ketone **9**. After 3N aqueous NaOH (2 mL) was added and stirred for 30 min, the reaction mixture was diluted with EtOAc (1 mL) and the organic phase was colleted. The aqueous phase was extracted with EtOAc (2 x 2 mL), and the combined organic phases were washed with brine (2 mL), dried over MgSO4, and concentrated *in vacuo* to yield a yellow powder. This material was directly used in the next step.

In a round-bottom flask equipped with a magnetic stir bar, the crude residue was dissolved in dry MeOH (0.85 mL) and CH_2Cl_2 (1.7 mL). Dimethylamine hydrochloride (61 mg, 0.75 mmol), 4Å molecular sieves (20 mg), and sodium cyanoborohydride (2.0

mg, 0.03 mmol) were added in this order, in on portion respectively. Triethylamine (0.10 μL, 0.075 mmol) was added and the yellow suspension was stirred at rt for 48 h until TLC analysis indicated complete consumption of the starting material. The mixture was diluted with CH_2Cl_2 (5 mL) and aqueous $NaHCO_3$ (10 mL) was added. The organic phase was isolated, and the aquoeous phase was extracted with CH_2Cl_2 (3 x 5 mL). The combined organic phases were washed with brine (5 mL), dried over $MgSO_4$, and concetrated *in vacuo* to afford a yellow powder. This material was directly used in the next step.

In a round-bottom flask equipped with a magnetic stir bar, the yellow powder was suspended in CH_2Cl_2 (1.25 mL). Triisopropylsilane (51 μL, 0.25 mmol) was added in one portion, and TFA (192 μL, 2.5 mmol) was added via syringe dropwise over 30 sec. On addition, the suspension turned into a bright yellow solution, which was stirred at rt for 30 min. The reaction mixture was diluted with CH_2Cl_2 (5 mL) and concentrated *in vacuo* to yield a yellow-brown residue. The crude product was purified by preparative reverse-phase HPLC (Alltech Alltima C18 10u, 250 mm X ID 22 mm, 5 – 95% acetonitrile/water gradient, flow rate = 20 mL/min) to afford the desired product as a bright yellow poder (6.8 mg, 43% yield) after lyopholization.

^{1}H NMR (600 MHz, *d$_6$*-DMSO): δ = 11.20 (brs, 1H), 9.95 (brs, 1H), 9.03 (d, 1H, J = 7.5 Hz), 7.92 (brs, 3H), 7.80 (d, 1H, J = 8.5 Hz), 7.62 (dt, 1H, J = 0.8 Hz, 7.3 Hz), 7.33 (t, 1H, J = 7.3 Hz), 4.65 (m, 2H), 4.50 (dd, 1H, J = 7.8 Hz, 13.9 Hz), (3.69 (dd, 2H, J = 8.4 Hz, 15.0 Hz), 3.02 (dd, J = 11.3 Hz, 14.9 Hz), 2.93 (m, 2H), 2.94 (t, 1H, J = 12.8 Hz), 2.69 (s, 6H), 2.46 (m, 1H), 2.35 (m, 1H), 2.11 (q, 1H, J = 7.6), 1.60 (q, 1H, J = 12.0 Hz), 1.47 (q, 1H, J = 12.0 Hz) ppm.

^{13}C NMR (125 MHz, *d$_6$*-DMSO): δ = 171.2, 169.6, 158.3, 158.0, 143.4, 141.3, 149.5, 131.0, 128.4, 125.1, 124.7, 120.9, 120.8, 119.9, 119.4, 110.1, 116.1, 66.7, 42.9, 41.6, 36.4, 27.9, 26.3, 23.1, 22.0 ppm.

IR (KBr) ν = 3432, 2963, 2376, 2346, 2094, 1687, 1459, 1326, 1279, 1261, 1202, 1132, 1089, 800, 722 cm^{-1}.

HRMS (*m/z*): Calculated: 405.2291 (for [$M+H^+$] $C_{24}H_{29}N_4O_2$); found: 405.2302.

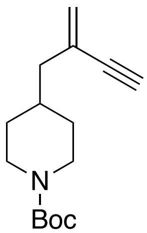

***tert*-butyl 4-(2-methylenebut-3-yn-1-yl)piperidine-1-carboxylate (15)** The procedure reported by Klusener and co-workers was used with modification.[3] *n*-BuLi (32.2 mL, 50.9 mmol, 1.6 M in hexanes) was added dropwise over 10 min to a three-neck flask equipped with an internal thermometer and an addition funnel containing 2-methyl-1-buten-3-yne (5.58 , 2.18 mL, 23.4 mmol) that had been cooled to -78 °C. *t*-BuOK (50.9 mL, 50.9 mmol, 1.0 M in THF) was added to the yellow suspension over 15 min, causing the reaction to slowly turn orange. The reaction was stirred for 30 min at -78 °C then for 15 min at 0 °C, which caused the reaction to turn yellow. The reaction was then cooled to -20 °C and LiBr (4.46 g, 50.9 mmol) was added as a solution in THF (15 mL) over 5 min causing the reaction to turn back to orange. After 15 min, Boc-4-bromopiperidine (5.57 g, 4.64 mL, 23.4 mmol) was added as a solution in THF (6.0 mL) over 5 min and the reaction was allowed to stir for 15 min at -20 °C then 30 min at 0 °C. The reaction was quenched with slow addition (over ~5 min) of saturated ammonium chloride (20 mL), extracted with Et_2O (3 x 90 mL), dried over MgSO4 and concentrated *in vacuo*. The residue was purified via flash column chromatography (10% EtOAc/pentane), yielding an orange semi-solid (3.87 g, 66%).

TLC R_f = 0.21 (5% EtOAc/pentane), one brown spot in *p*-anisaldehyde stain.

^{1}H NMR (500 MHz, $CDCl_3$): δ = 5.44 (d, J = 1.6 Hz, 1H), 5.25 (d, J = 1.1 Hz, 1H), 4.06 (s, 2H), 2.87 (s, 1H), 2.68 (s, 2H), 2.06 (d, J = 7.0 Hz, 2H), 1.69-1.77 (m, 1H), 1.63-1.69 (d, J = 13.2 Hz, 2H), 1.43 (s, 9H), 1.05 (qd, J = 12.5, 4.0 Hz, 2H) ppm.

^{13}C NMR (125 MHz, CDCl3): δ = 155.0, 128.8, 124.5, 84.1, 79.4, 77.2, 44.2, 34.6, 31.8, 28.62, 28.61 ppm.

IR (KBr) ν = 3227, 2975, 2929, 2850, 1692, 1421, 1365, 1277, 1241, 1165, 1116, 1072 cm^{-1}.

HRMS (*m/z*): Calculated: 272.1626 (for [M+Na$^+$] $C_{15}H_{23}NNaO_2$); found: 272.1615.

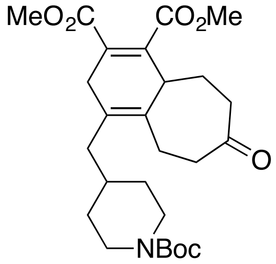

Dimethyl 1-((1-(*tert*-butoxycarbonyl)piperidin-4-yl)methyl)-7-oxo-4a,5,6,7,8,9-hexahydro-2*H*-benzo[7]annulene-3,4-dicarboxylate (16) VCP **1** (15 µL, 0.10 mmol) was added via a syringe to a stirring solution of [($C_{10}H_8$)Rh(COD)]SbF_6 (2.9 mg, 0.0050 mmol) in DCE (0.25 mL) in an oven-dried flask under an inert atmosphere at rt. A solution of enyne **15** (30 mg, 0.12 mmol) in DCE (0.25 mL) was then immediately added in a single portion via syringe, upon which the homogeneous yellow reaction solution became dark orange in color. As soon as TLC analysis confirmed consumption of enyne **15** (45 min), DMAD (14 µL, 0.11 mmol) was added in a single portion via a syringe. After the reaction had stirred at rt for an additional 15 min, TLC analysis indicated consumption of all [5+2] intermediates and the reaction mixture was treated with one drop of water and HCl (25 µL of a 1% solution in EtOH). When [5+2]/[4+2] intermediates had converged to a single orange spot on TLC (*p*-anisaldehyde stain), the reaction solution was passed through a short pad of silica gel (EtOAc eluent) and concentrated. The crude product was purified using flash column chromatography (40% EtOAc/pentane) to furnish bis-ene cycloadduct **16** (36 mg, 75%) as a thick, sticky, colorless oil.

TLC R_f = 0.39 (60% EtOAc/pentane), UV-active, one orange spot in *p*-anisaldehyde stain.

^{1}H NMR (400 MHz, $CDCl_3$): δ = 4.20-4.00 (br m, 2H), 3.79 (s, 3H), 3.77 (s, 3H), 3.24-3.16 (m, 1H), 2.93-2.80 (m, 3H), 2.76 (td, Jt = 12.8 Hz, Jd = 3.1 Hz, 1H), 2.69-2.53 (m, 3H), 2.48 (ddd, J = 13.1, 6.8, 2.5 Hz, 1H), 2.39 (ddd, J = 16.6, 11.7, 5.1 Hz, 1H), 2.22-2.10 (m, 2H), 2.06 (dd, J = 13.8, 7.5 Hz, 1H), 1.93 (dd, J = 14.0, 6.4 Hz, 1H), 1.64-1.48 (br m, 3H), 1.45 (s, 9H), 1.41-1.27 (m, 1H), 1.17-1.01 (br m, 2H) ppm.

^{13}C NMR (125 MHz, CDCl3): (100 MHz, CDCl3): δ = 213.1, 168.3, 167.9, 154.9, 137.5, 132.2, 131.8, 127.3, 79.5, 52.5, 52.5, 45.4, 44.1 (br), 42.3, 42.2, 38.6, 34.6, 32.5, 32.2, 31.6, 28.6, 26.8 ppm.

IR (KBr) ν = 2930 (m), 1721 (s), 1690 (s), 1427 (m), 1365 (w), 1265 (s), 1165 (m), 1113 (w), 1078 (w), 1021 (w), 959 (w), 864 (w), 769 (w), 731 (w) cm^{-1}.

HRMS (*m/z*): Calculated: 498.2468 (for [M+Na$^+$] $C_{26}H_{37}NNaO_7$); found: 498.2466.

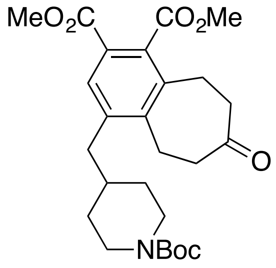

Dimethyl 4-((1-(tert-butoxycarbonyl)piperidin-4-yl)methyl)-7-oxo-6,7,8,9-tetrahydro-5H-benzo[7]annulene-1,2-dicarboxylate (17) DDQ (805 mg, 3.55 mmol) was added to a vial containing **16** (375 mg, 0.79 mmol) as a solution in toluene (16.0 mL) and a magnetic stir bar. The vial was sealed with a screw cap and placed in an 80 °C oil bath for 2 hours. The reaction mixture was then cooled to rt and loaded directly onto a silica gel column and purified by column flash chromatography (1:2 EtOAc/pentane) to give the desired product as a red amorphous solid (296 mg, 79%).

TLC R_f = 0.52 (60% EtOAc/pentane), UV-active, one red/orange spot in *p*-anisaldehyde stain.

^{1}H NMR (500 MHz, CDCl3): δ = 7.70 (s, 1H), 4.00-4.21 (bs, 2H), 3.94 (s, 3H), 3.98 (3, 3H), 2.99-3.04 (m, 2H), 2.86-2.91 (m, 2H), 2.54-2.70 (m, 8H), 1.49-1.62 (m, 3H), 1.45 (s, 9H), 1.14-1.26 (m, 2H) ppm.

^{13}C NMR (125 MHz, CDCl3): δ = 209.3, 170.1, 166.1, 154.9, 145.0, 139.1, 138.6, 133.5, 131.1, 125.6, 79.6, 52.92, 52.91, 52.73, 46.0, 43.9, 43.6, 41.0, 38.0, 32.18, 32.17, 28.60, 28.55, 26.6, 24.4 ppm.

IR (KBr) ν = 2930, 2863, 1725, 1690, 1431, 1278, 1164, 1114 cm^{-1}.

HRMS (*m/z*): Calculated: 496.2311 (for [M+Na$^+$] $C_{26}H_{35}NNaO_7$); found: 496.2311.

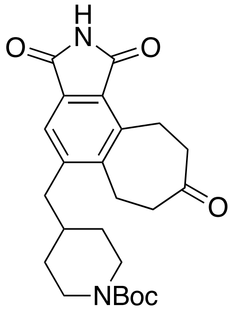

***tert*-butyl 4-((1,3,8-trioxo-1,2,3,6,7,8,9,10-octahydrocyclohepta[*e*]isoindol-5-yl)methyl)piperidine-1-carboxylate (18)** To a vial containing **17** (49 mg, 0.10 mmol) in ethanol (2.0 mL) was added potassium hydroxide (0.52 mL of a 1.0 N aqueous solution), producing a red homogeneous solution. The vial was capped and heated in an 80 °C oil bath for 4 h. After cooling to rt, the solution was treated with hydrochloric acid (5.2 mL of a 0.1 N aqueous solution), and this yellow mixture was poured into a separatory funnel contianing H_2O and EtOAc (30 mL each). The layers were separated, the aqueous layer was extracted with EtOAc (3 x 30 mL) and the combined organic lyaers were washed with brine and concentrated to a dark yellow-brown solid. This solid (12 mg) was carried on without further purification and dissolved in acetic anhydride (21.6 mL) and heated to 50 °C for 18 h. Excess acetic anhydride was removed *in vacuo*, and the residue contianing the crude anhydride was re-dissolved in dry DMF (0.5 mL) under a nitrogen atmosphere and treated with MeOH/HMDS (110 µL of a 1:2 molar mixture, pre-stirred for 5 minutes at rt in an oven-dried flask under nitrogen).[4] The reaction vessel was heated in an 80 °C oil bath for 3 h. After the mixture had cooled to rt, EtOAc was added (20 mL) and the organic phase was washed with brine and water, dried over Na_2SO_4, and concentrated to an oil. The crude product was purified via flash column chromatography (50% EtOAc/pentane) to give **18** (17 mg, 41% yield over three steps) as a yellow amorphous solid.

TLC R_f = 0.50 (50% EtOAc/pentane), UV-active, one red spot in *p*-anisaldehyde stain.

^{1}H NMR (500 MHz, $(CD_3)_2CO$, 45 ºC): δ = 7.56 (s, 1H), 4.06 (br d, J = 12 Hz, 2H), 3.69-3.62 (m, 2H), 3.25-3.17 (m, 2H), 2.87 (d, J = 7.5 Hz, 2H), 2.75-2.52 (m, 6H), 1.84-1.73 (m, 1H), 1.64 (br d, J = 13 Hz, 2H), 1.43 (s, 9H), 1.28-1.18 (m, 2H) ppm.

^{13}C NMR (125 MHz, $CDCl_3$): δ = 209.3, 169.0, 167.7, 164.9, 147.4, 144.9, 141.9, 131.1, 127.0, 124.3, 79.7, 43.5, 43.4, 41.7, 38.0, 32.2, 30.4, 28.6, 24.2, 22.4 ppm.

IR (KBr) ν = 3233, 2929, 1764, 1717, 1427, 1366, 1335, 1303, 1283, 1246, 1161, 1115, 961, 916, 862, 754, 732, 640 cm^{-1}.

HRMS (*m/z*): Calculated: 449.2053 (for [$M+Na^+$] $C_{24}H_{30}NNaO_5$); found: 449.2058.

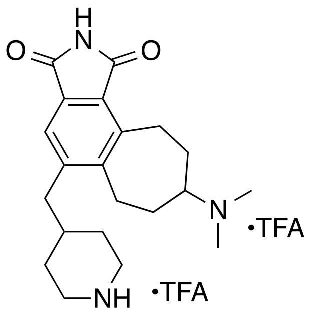

8-(dimethylamino)-5-(piperidin-4-ylmethyl)-7,8,9,10-tetrahydrocyclohepta[e]isoindole-1,3(2H,6H)-dione **(19)** A solution of ketone **18** in a 1:2 mixture of anhydrous MeOH/CH_2Cl_2 (3.5 mL) was treated with dimethylamine hydrochloride (86.0 mg, 1.06 mmol) and activated (via heated vacuum oven) powdered molecular sieves (50.0 mg) under Ar atmosphere. Et_3N (15 µL, 0.11 mmol) was added in one portion. After 48 h, **18** was consumed by TLC and saturated aqueous ammonium chloride (5.0 mL) and CH_2Cl_2 (5.0 mL) were added. The organic layer was collected and the aqueous layer was treated with aqueous 2 M NaOH soluiton (5.0 mL). The asqueous layer was extracted with CH_2Cl_2 (3 x 5.0 mL) and the combined organic layers were washed with brine, dried over Na_2SO_4, and concentrated to yield a yellow solid. The crude product was purified by column chomratography on silica (20:1 to 10:1 DCM:MeOH), yielding the amine (11 mg) as a colorless amorphous solid, which was taken on to the next step.

A solution of the amine (7.50 mg, 0.016 mmol) in anhydrous CH_2Cl_2 (0.82 mL) under Ar atmosphere was treated with triisopropylsilane (34 µL, 0.16 mmol) in one portion, causing the reaction mixture to become a cloudy white suspension. TFA (0.13 mL, 1.65 mmol) was next added dropwise over 30 sec, which caused the solution to become colorless. After 30 min, the stir bar was removed and CH_2Cl_2 (5.0 mL) was

added, then the solution was concentrated *in vacuo* to yield a yellow oil. The crude oil was then purified using preparative reverse phase HPLC (MeCN with 0.1% TFA / H_2O with 0.1% TFA: 5% to 95% gradient) to give the desired product as a colorless amorphous solid after lyopholization.

^{1}H NMR (600 MHz; CD_3OD): δ 7.53 (s, 1H), 4.53-4.49 (m, 1H), 3.70-3.65 (m, 1H), 3.40-3.34 (m, 3H), 2.97 (d, J = 0.6 Hz, 3H), 2.83 (s, 6H), 2.80-2.66 (m, 3H), 2.44-2.37 (m, 2H), 1.89-1.84 (m, 3H), 1.59-1.44 (m, 4H).

^{13}C NMR (101 MHz, CD_3OD, 50ºC): δ = 171.6, 170.4, 149.1, 144.7, 143.1, 132.6, 128.7, 124.7, 69.8, 45.2, 41.4, 40.2, 36.3, 29.81, 29.77, 28.2, 27.6, 26.0, 23.8 ppm.

IR (KBr) ν = 3416, 3052, 2851, 2746, 1760, 1713, 1678, 1470, 1428, 1377, 1343, 1318, 1202, 1133, 994, 975, 838, 801, 750, 722, 666 cm^{-1}.

HRMS (*m/z*): Calculated: 356.2333 (for $[M+H^+]$ $C_{21}H_{30}N_3O_2$); found: 356.2324.

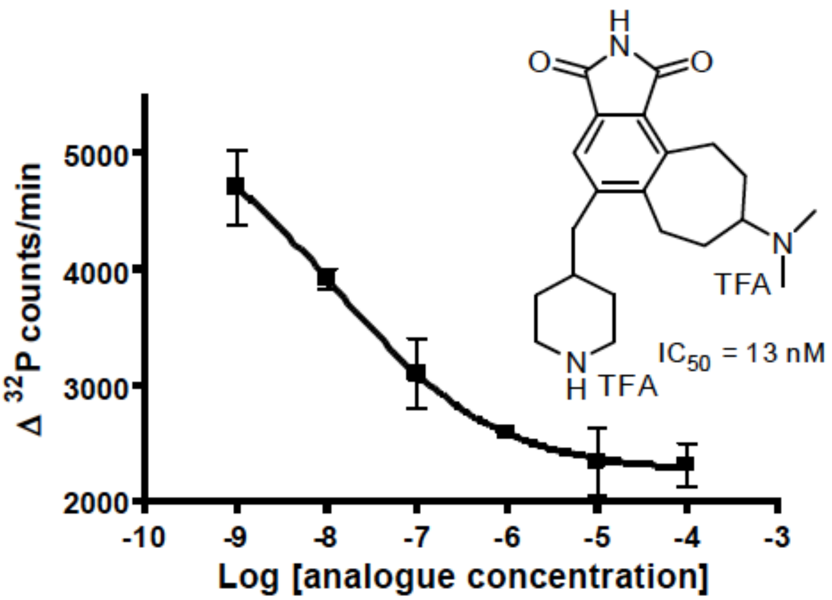

PKC Inhibition Assay

A PKC inhibition assay was performed following the protocol developed by Kikkawa and coworkers.[5] The assay can be scaled as desired. Histone solution (0.33 mg/mL in 20 mM Tris buffer) was prepared from a stock solution (33 mg/mL, stored frozen). Phosphatidylserine (PS) vesicles were prepared by the addition of PS (10 mg/mL in chloroform, 240 μL) and dioleoylglycerol (10 mg/mL in ethanol, 40 μL) to a glass vial followed by removal of the chloroform under a stream of nitrogen. To the neat PS was added chilled 20 mM Tris assay buffer (2 mL) and the resulting mixture was sonicated (Branson Sonifier 250, power = 3, 30% duty cycle) four times for 30 sec with a 30 sec interval between sonications. The resulting milky suspension was stored on ice. "Stop" solution refers to a solution of 200 mM EDTA and 200 mM ATP in 20 mM Tris buffer. Whatman filter papers were cut in 1 x 1 inches in dimension and labeled with tube number in pencil. An "Autogo" solution was prepared by mixing 27 μL of 3.75 mM ATP (in 20 mM Tris), 100 μL of 1.0 M MgCl2, 872 μL of 20 mM Tris buffer, and 1.3 μL of [γ-32P] ATP (total volume is 1 mL). "Autogo" solution should be kept in the hot hood all the time. "Cocktail 1" was prepared by mixing 70 μL of 10 mM Ca^{2+},350 μL of 20 mM Tris, and 70 μL of histone solution (0.33 mg/mL in 20mM Tris). "Cocktail 2" was prepared by mixing 310 μL of 10 mM Ca^{2+}, 930 μL of 20 mM Tris, 310 μL of the histone solution, and 310 μL of the PS solution (added only once the assay is ready to be run).

To each eppendorf tube, 10 μL of PKC solution and10 μL of the inhibitor solution were added. After addition of the inhibitor, each tube was vortexed. For negative controls, 70 μL of cocktail 1 was added to tubes 1 and 2. To the rest of the tubes were added 60 μL of cocktail 2. All the tubes were then transferred to the hot hood, and 20 μL of "Autogo" solution was added to each tube and vortexed immediately. A start countdown of 7 min was initiated when "Autogo" was added to the first tube. Addition to all the tubes should be completed within 7 min. When the countdown is over, "Stop" solution was added to each tube and vortexed. The interval between the addition of "Autogo" and "Stop" solution should be 7 min for each tube.

Contents of each tube was transferred to the piece of pre-labeled Whatman filter paper and the paper was washed by soaking them and swirling in a beaker filled with 300 mL of tap water. Once all the filter papers are soaked in the beaker, the supernatant was carefully drained (to the hot hood drain) and the papers were rinsed by soaking them in 300 mL of fresh tap water for 2 min. The water was decanted, and the papers were rinsed by soaking them in 300 mL of ethanol for 2 min. The ethanol should be recovered to a bottle in the hot hood (not to the drain). The papers were dried in the hot hood and each paper was transferred to a scintillation vial. The radiation was measured with the scintillation counter (Beckman LS 6000SC). The data set was analyzed using a least-square data-fitting algorithm in Prism 4 (Graphpad Software Inc.) to give an IC_{50} value (defined as the concentration of analogue required to inhibit 50% of PKC activity).

References:

1. Wender, P. A.; Williams, T. J. *Angew. Chem., Int. Ed.* **2002** , *41* , 4550-4553.
2. Bergman, J.; Venemalm, L. *J. Org. Chem.* **1992**, *57*, 2495-2497.
3. Klusener, P. A. A.; Kulik, W.; Brandsma, L. *J. Org. Chem*. **1987**, *52* , 5261-5266.
4. Davis, P. D.; Bit, R. A. *Tet. Lett.* **1990**, *31*, 5201-5204.
5. Kikkawa, U.; Takai, Y.; Minakuchi, R.; Inohara, S.; Nishizuka, Y. *J. Biol. Chem.* **1982**, *25*, 13341-13348.

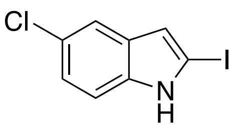
Cl
I
N
H

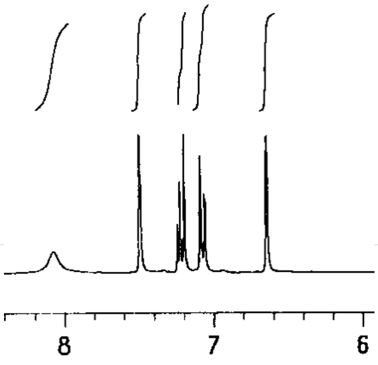
8
7
6

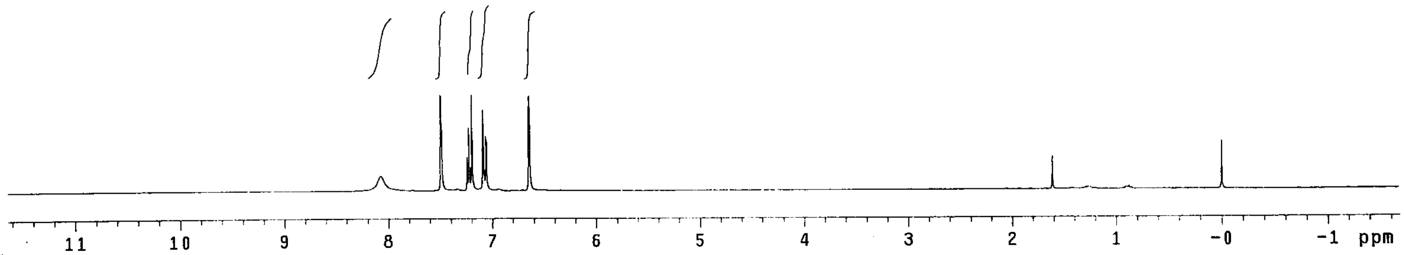
11
10
9
8
7
6
5
4
3
2
1
-0
-1 ppm

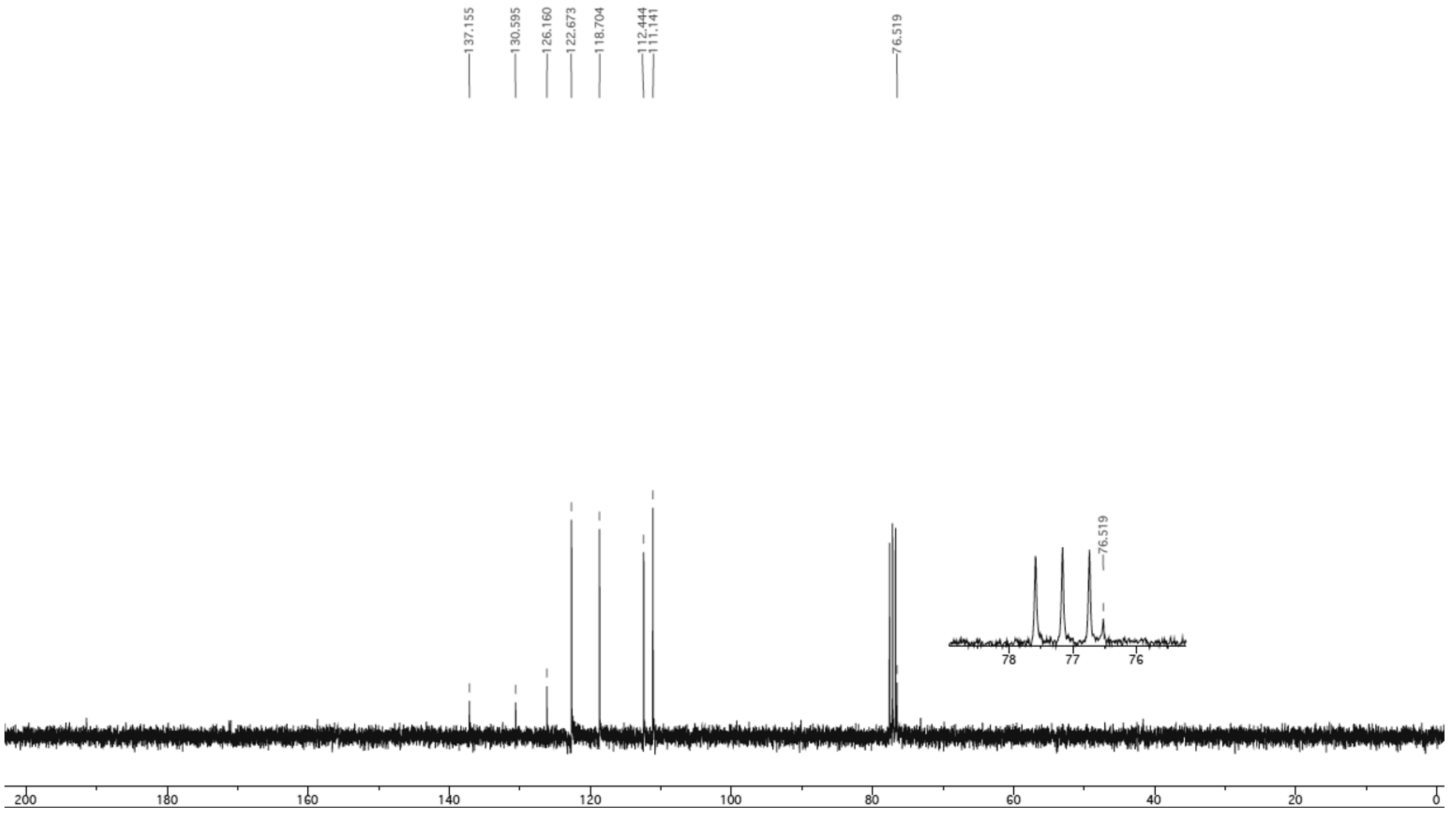
137.155
130.595
126.160
122.673
118.704
112.444
111.141
76.519
76.519
78
77
76
200
180
160
140
120
100
80
60
40
20
0

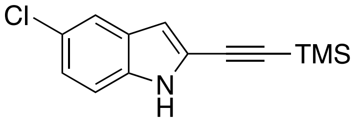
Cl
TMS
N
H

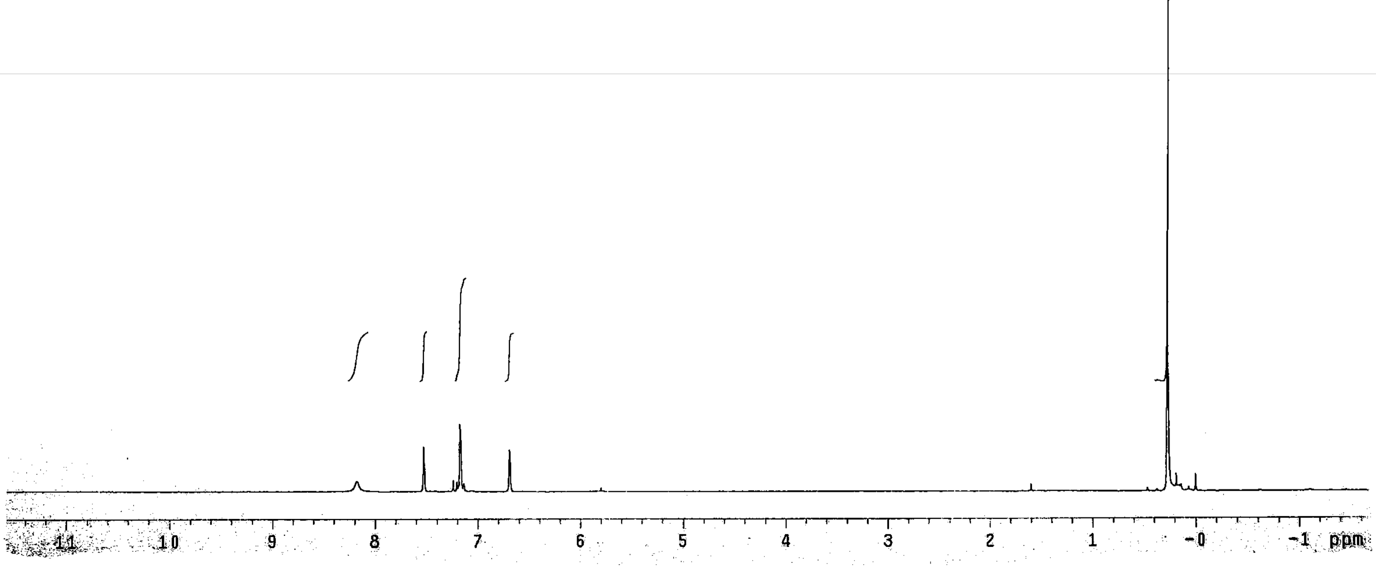
11 10 9 8 7 6 5 4 3 2 1 0 -1 ppm

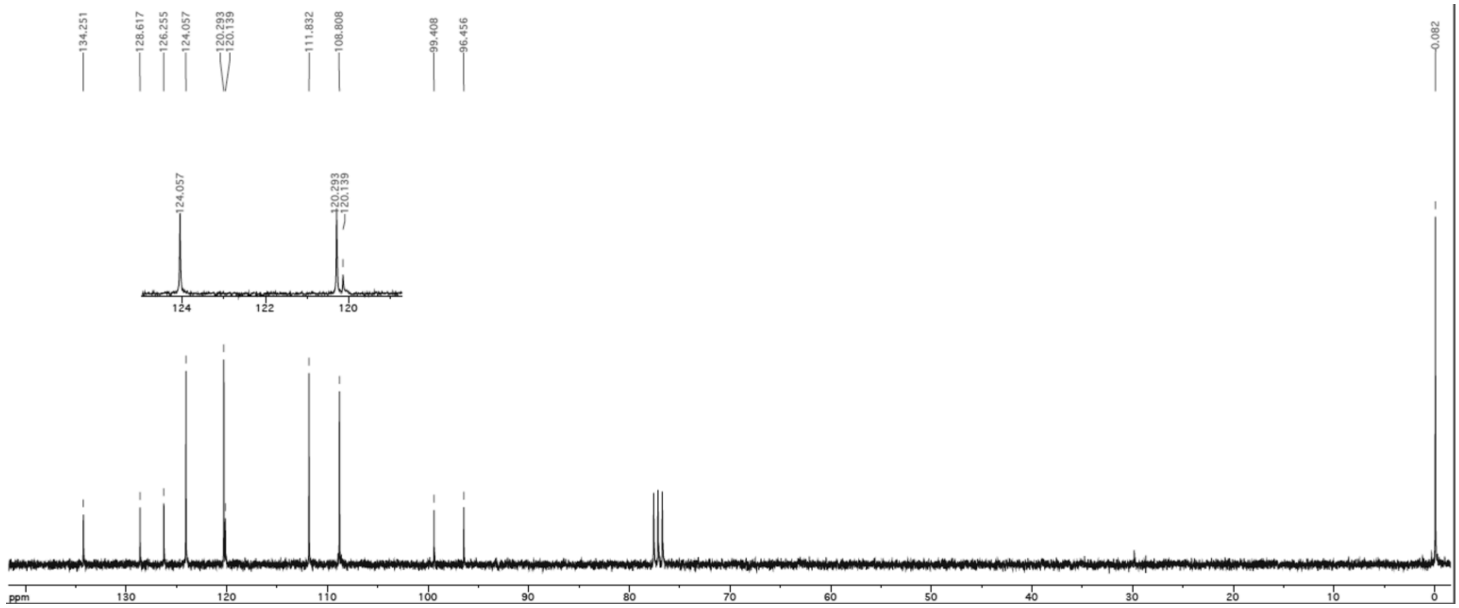
134.251
128.617
126.255
124.057
120.293
120.139
111.832
108.808
99.408
96.456
0.082
124.057
120.293
120.139
124 122 120
ppm 130 120 110 100 90 80 70 60 50 40 30 20 10 0

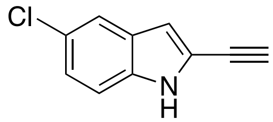
Cl
N
H

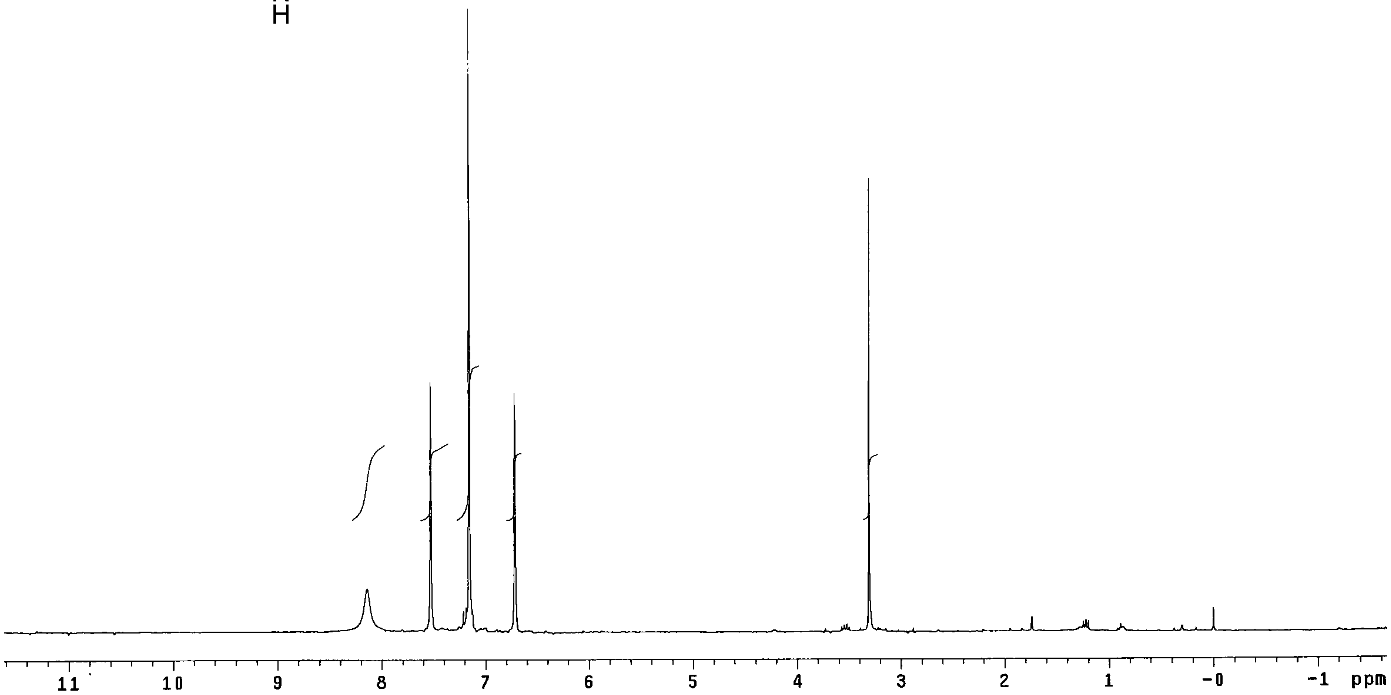
11 10 9 8 7 6 5 4 3 2 1 -0 -1 ppm

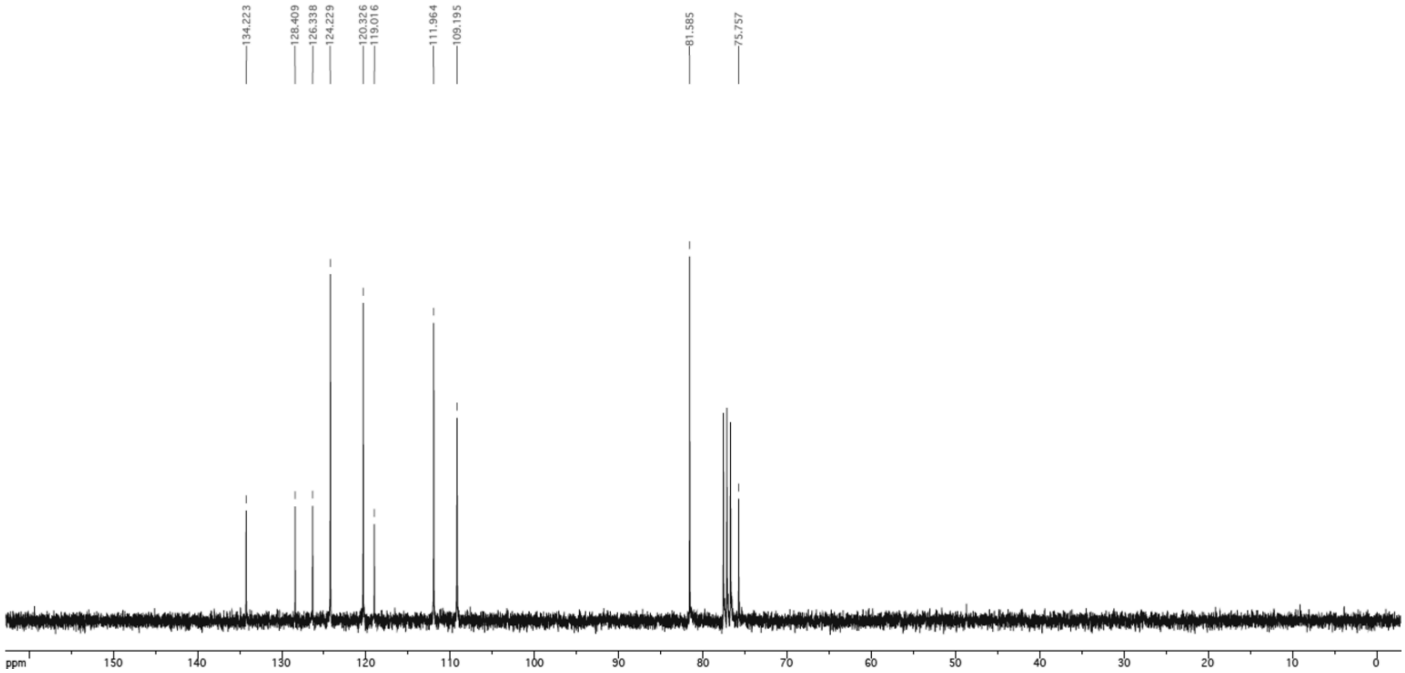
134.223
128.409
126.338
124.229
120.326
119.016
111.964
109.195
81.585
75.757
ppm 150 140 130 120 110 100 90 80 70 60 50 40 30 20 10 0

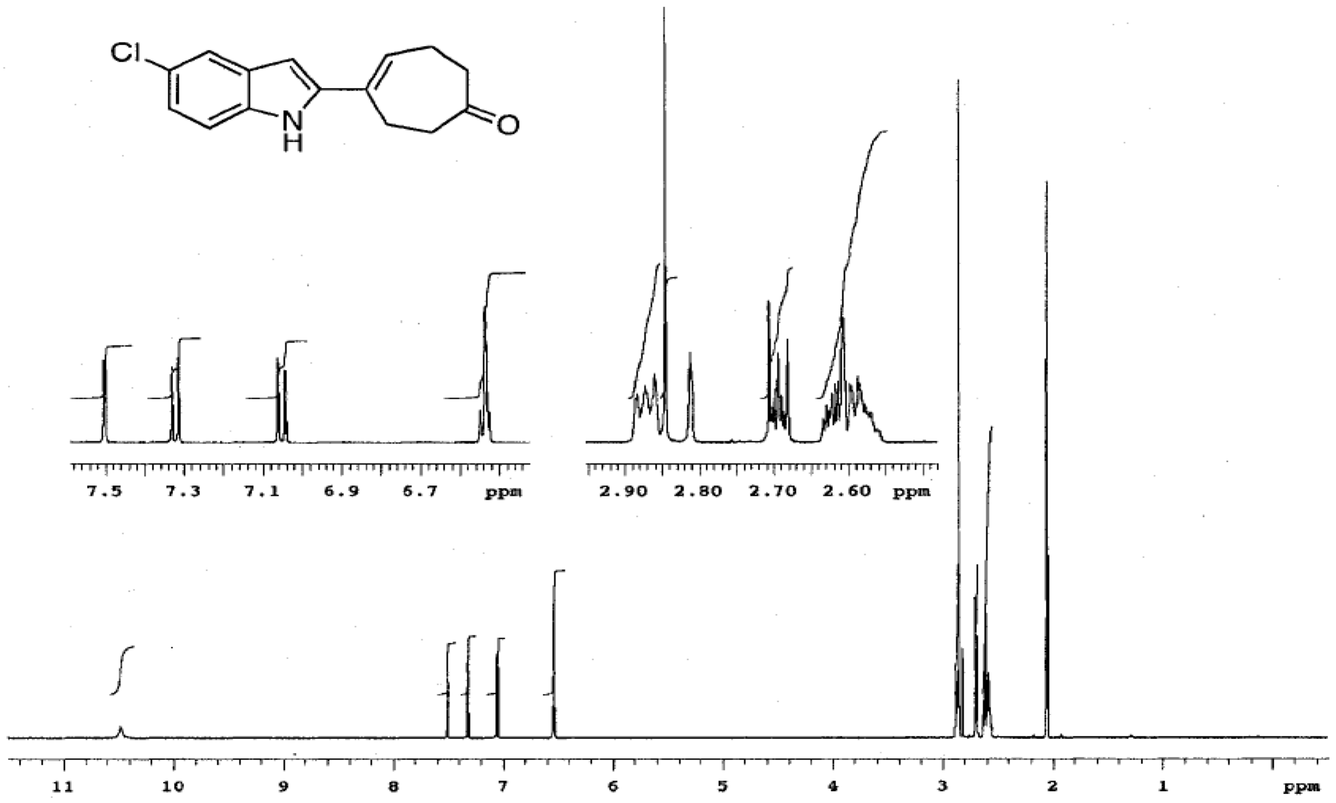
Cl
NH
O
7.5 7.3 7.1 6.9 6.7 ppm
2.90 2.80 2.70 2.60 ppm
11 10 9 8 7 6 5 4 3 2 1 ppm

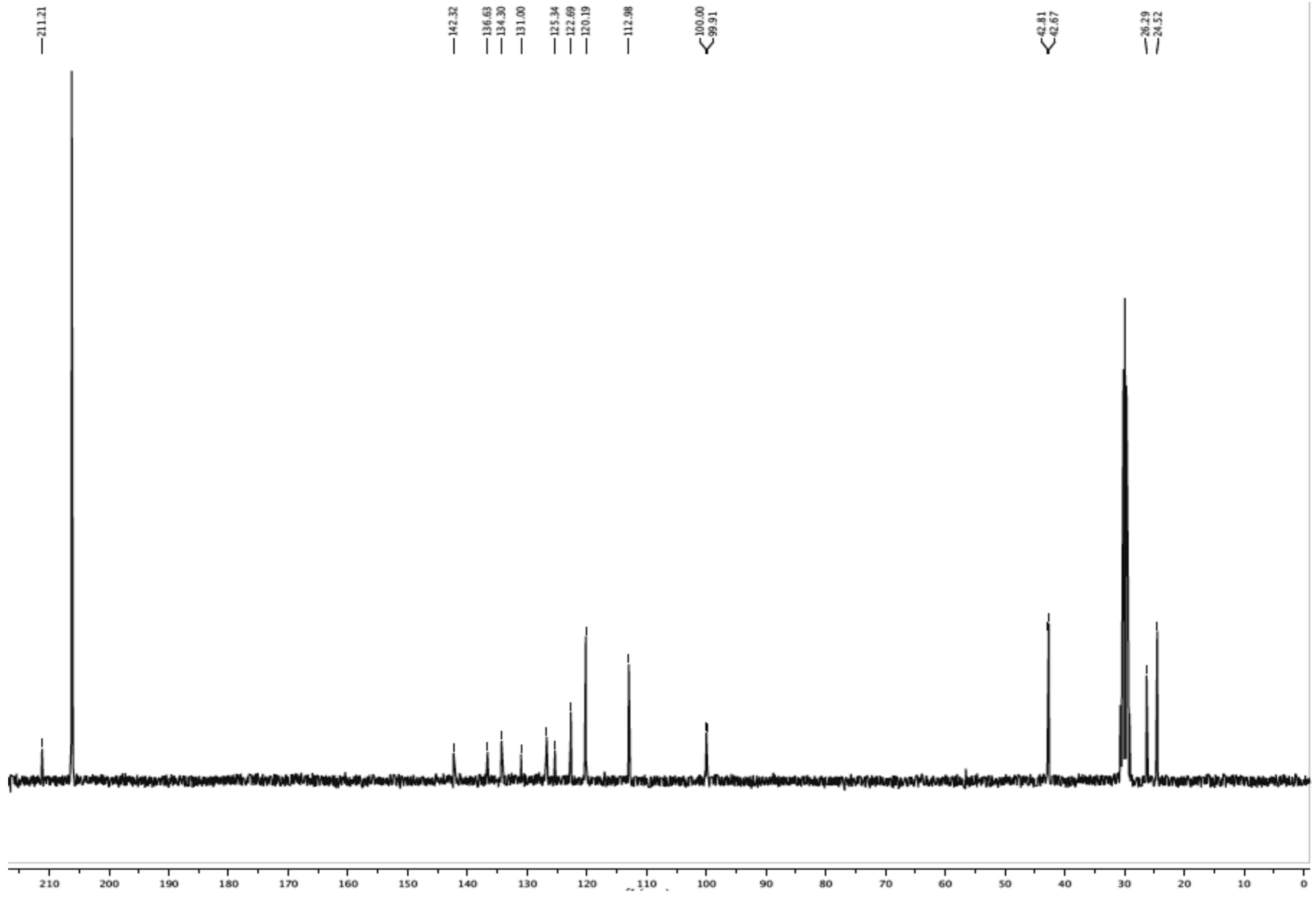
211.21
142.32
136.63
134.30
131.00
125.34
122.69
120.19
112.98
100.00
99.91
42.81
42.67
26.29
24.52
210 200 190 180 170 160 150 140 130 120 110 100 90 80 70 60 50 40 30 20 10 0

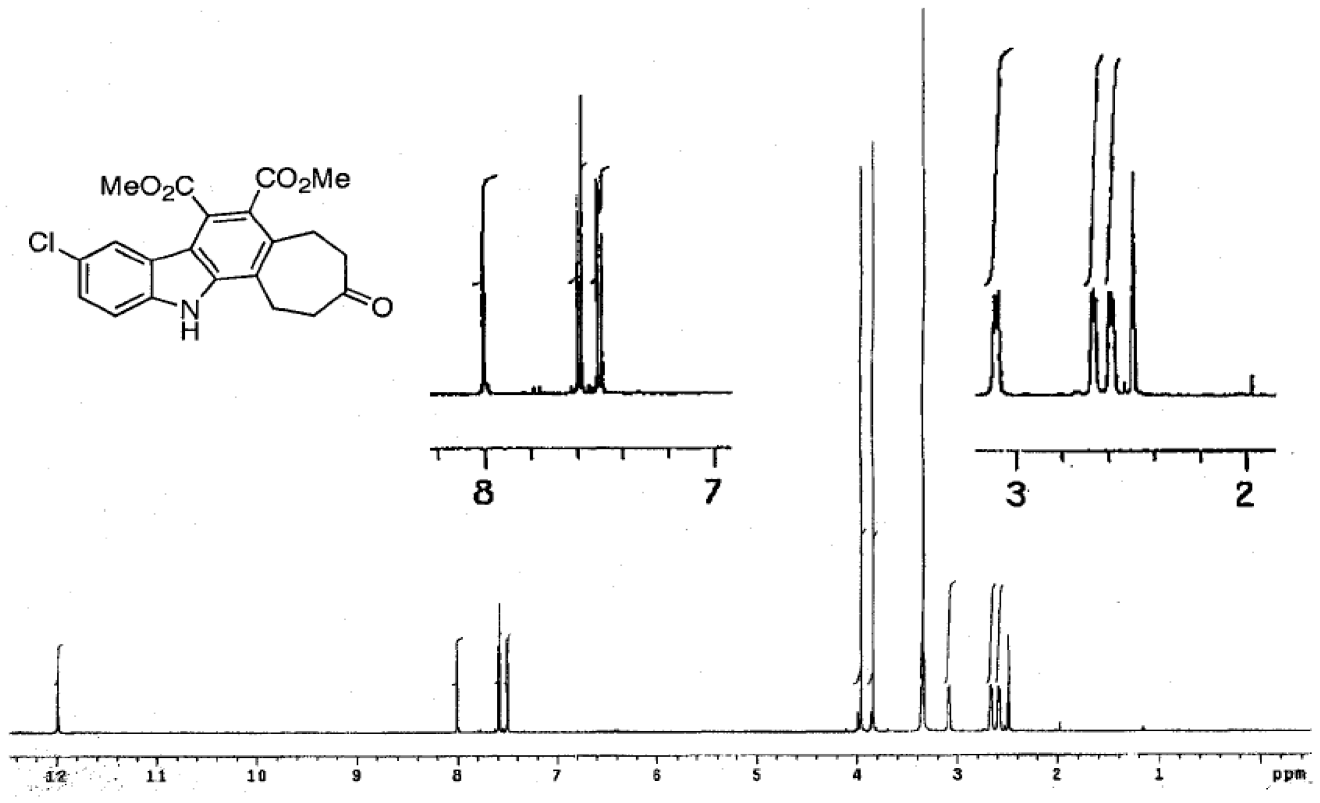
MeO2C
CO2Me
Cl
N
H
O
8
7
3
2
12
11
10
9
8
7
6
5
4
3
2
1
ppm

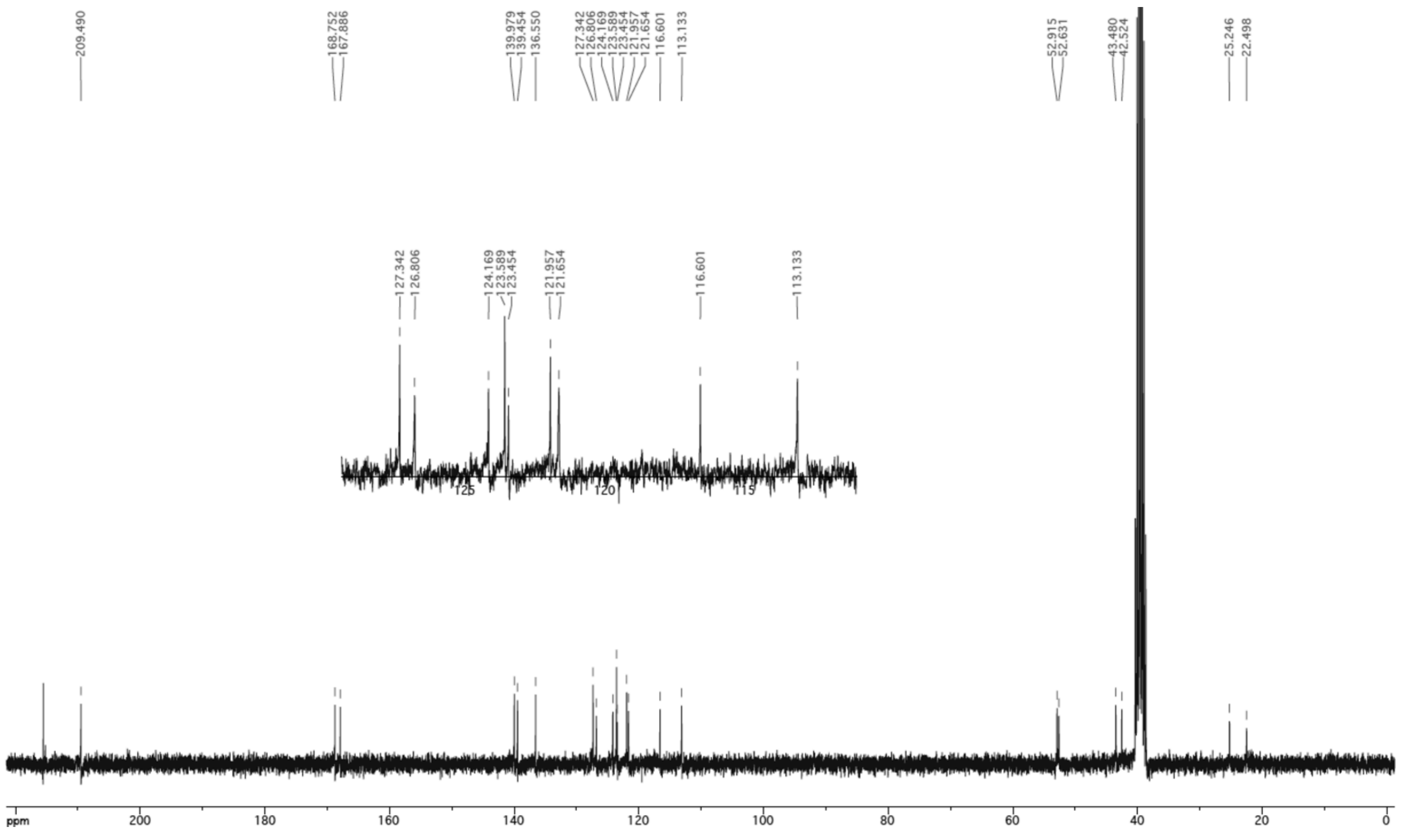
209.490
168.752
167.886
139.979
139.454
136.550
127.342
126.806
124.169
123.589
123.454
121.957
121.654
116.601
113.133
52.915
52.631
43.480
42.524
25.246
22.498
125
120
115
ppm
200
180
160
140
120
100
80
60
40
20
0

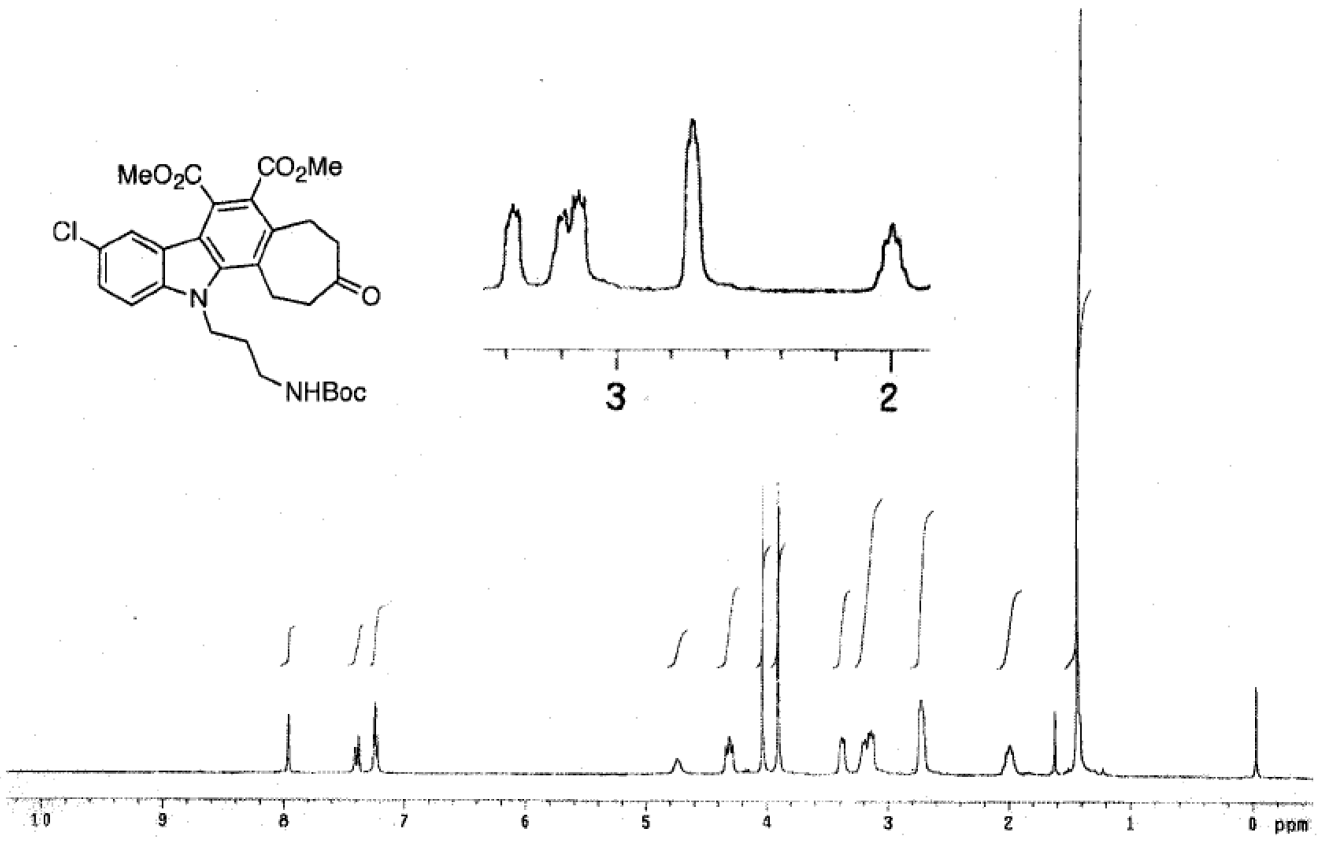
MeO2C
CO2Me
Cl
N
O
NHBoc
3
2
10 9 8 7 6 5 4 3 2 1 0 ppm

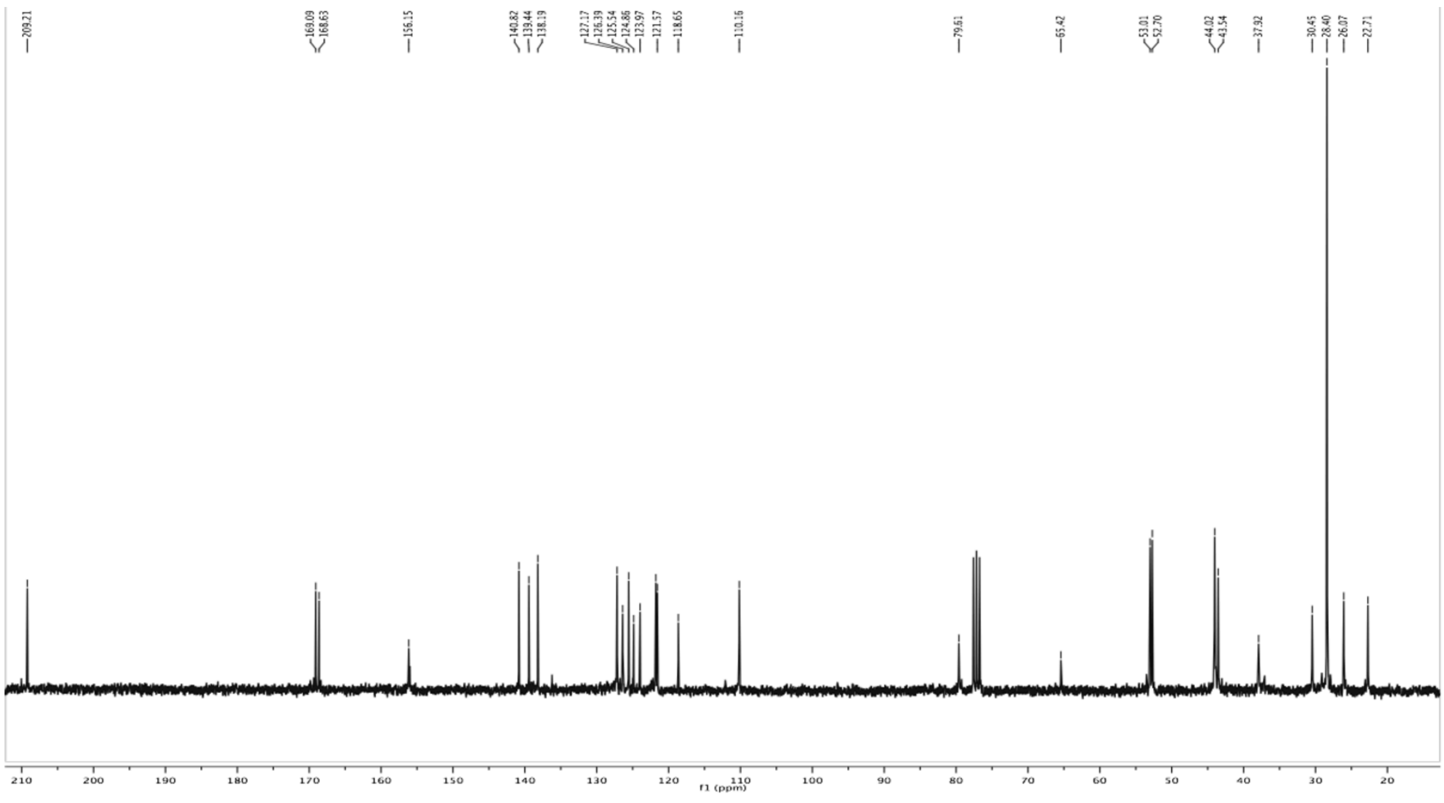
209.21
169.09
168.63
156.15
140.82
139.44
138.19
127.17
126.39
125.54
124.86
123.97
121.57
118.65
110.16
79.61
65.42
53.01
52.70
44.02
43.54
37.92
30.45
28.40
26.07
22.71
210 200 190 180 170 160 150 140 130 120 110 100 90 80 70 60 50 40 30 20
f1 (ppm)

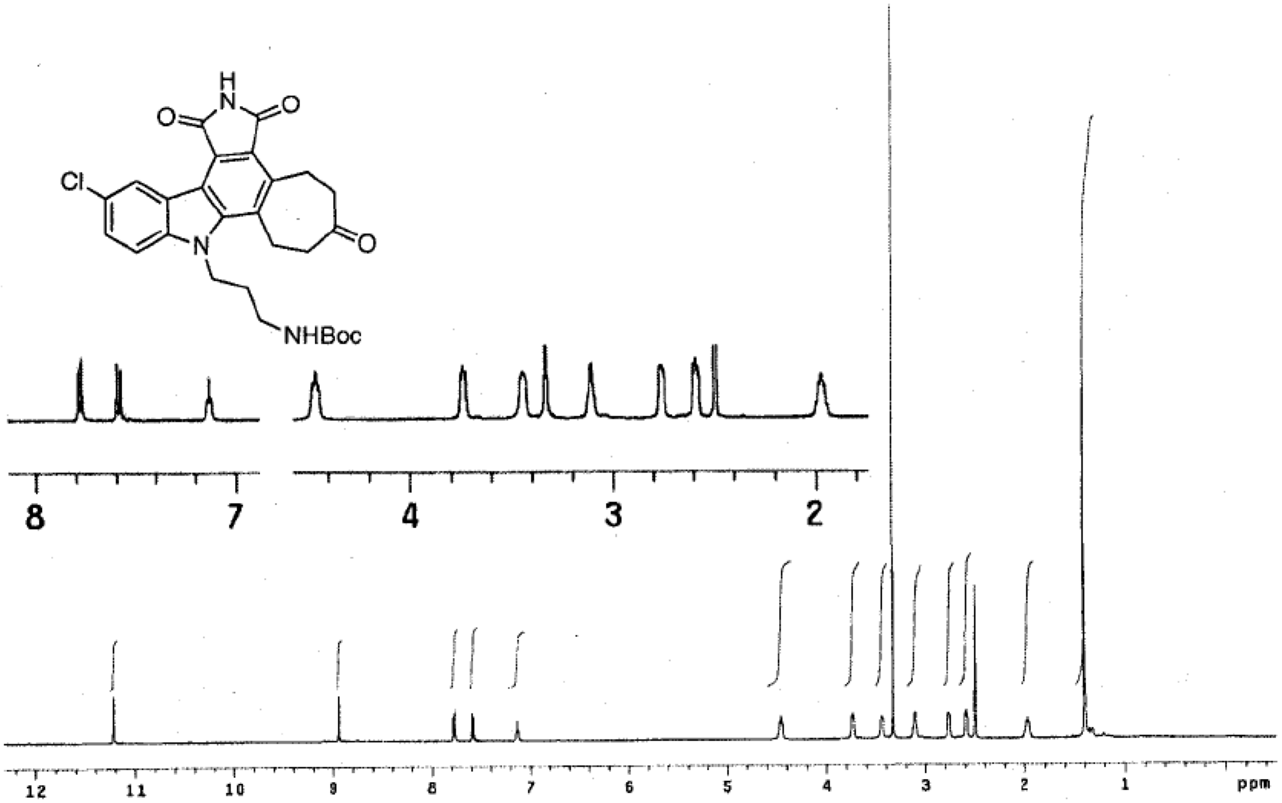
Cl
NHBoc
ppm

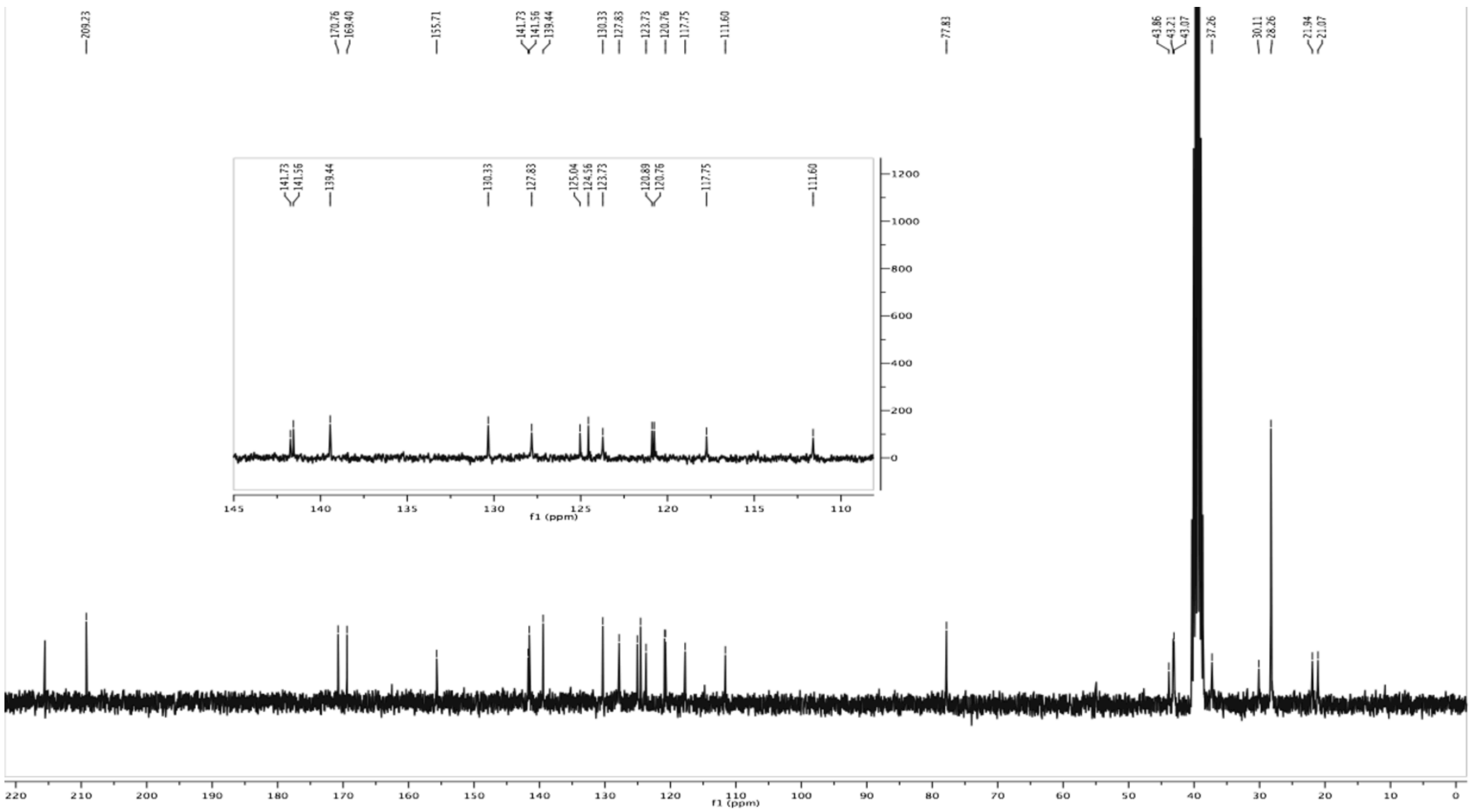
209.23
170.76
169.40
155.71
141.73
141.56
139.44
130.33
127.83
123.73
120.76
117.75
111.60
77.83
43.86
43.21
43.07
37.26
30.11
28.26
21.94
21.07
125.04
124.56
120.89
f1 (ppm)

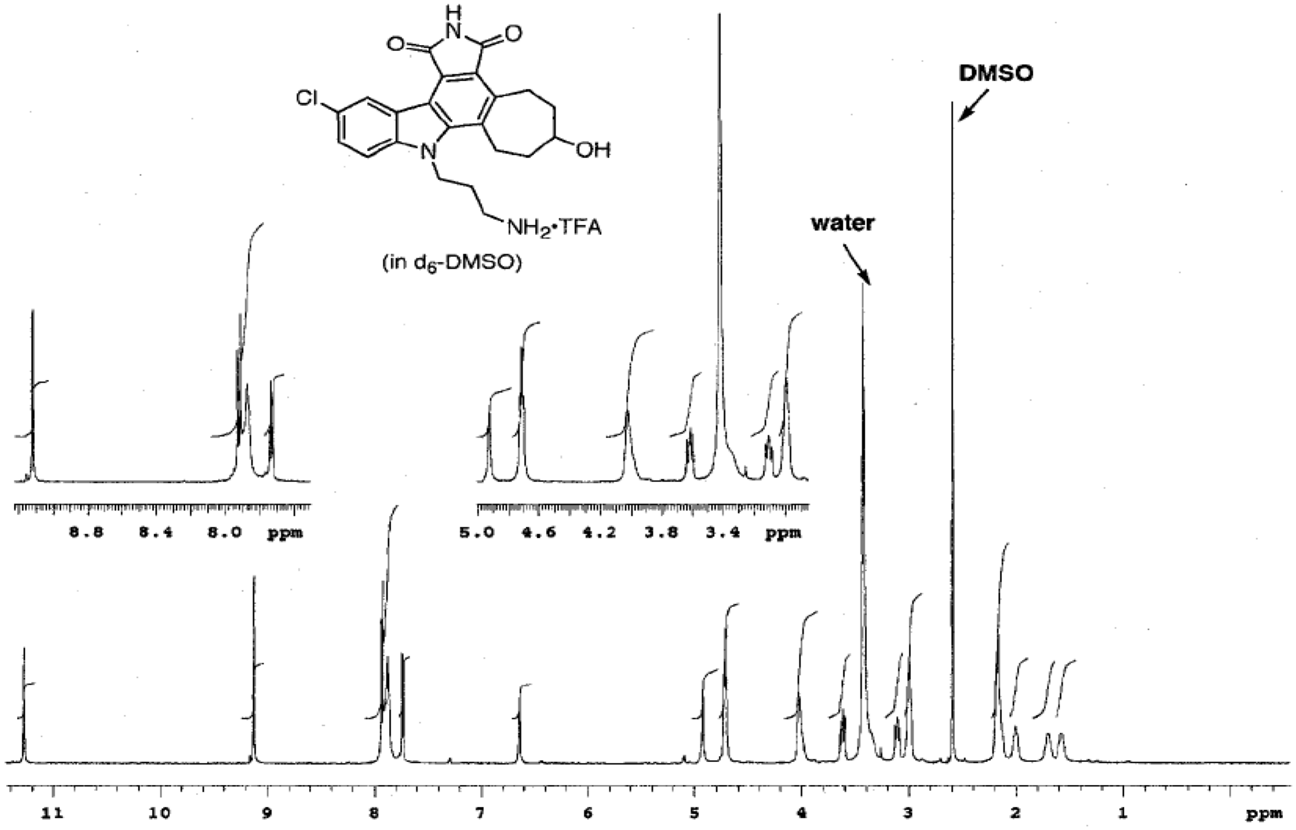

Cl
OH
NH2•TFA
(in d6-DMSO)
water
DMSO
ppm

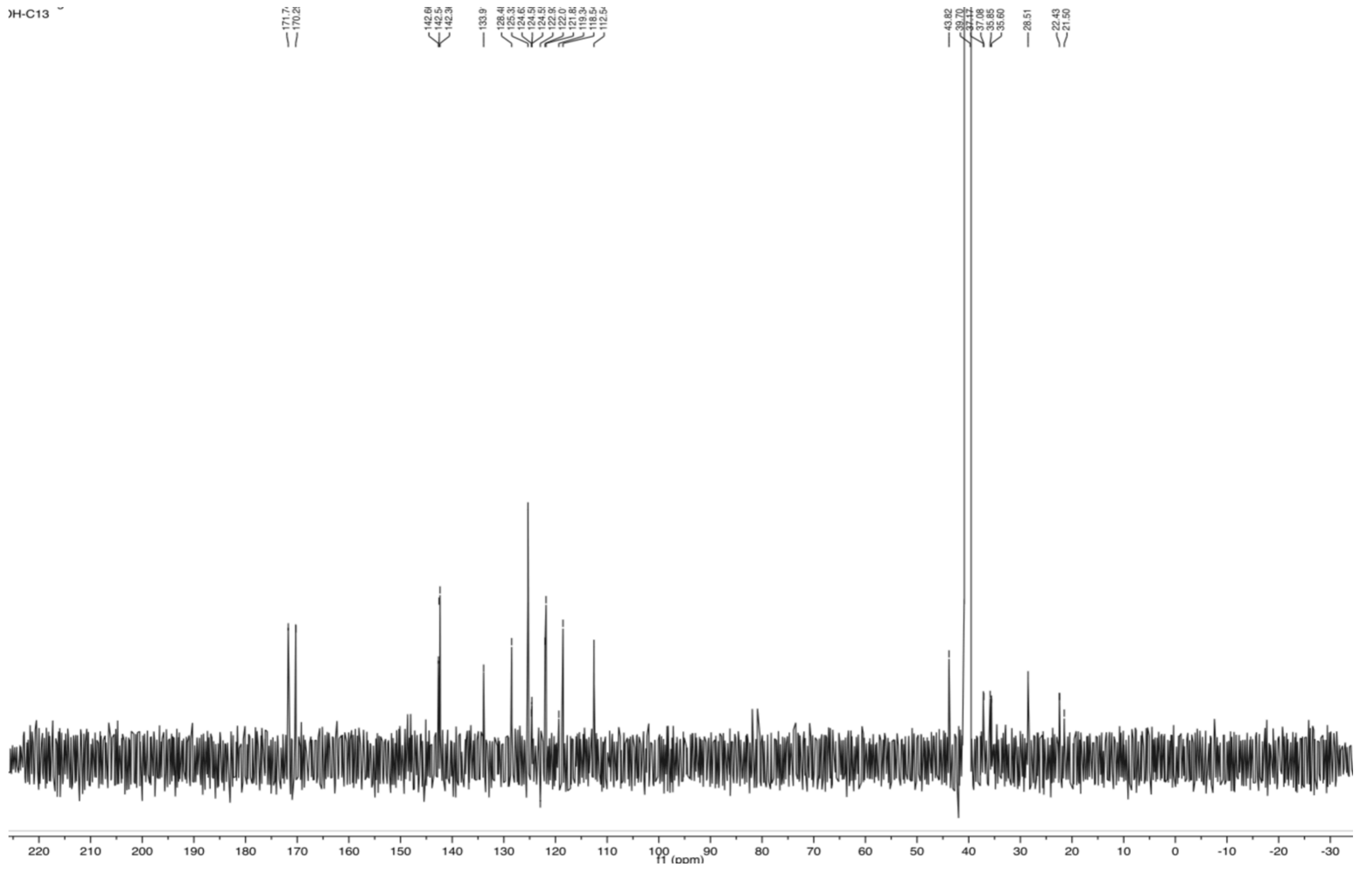

43.82
39.70
37.17
37.08
35.85
35.60
28.51
22.43
21.50
f1 (ppm)

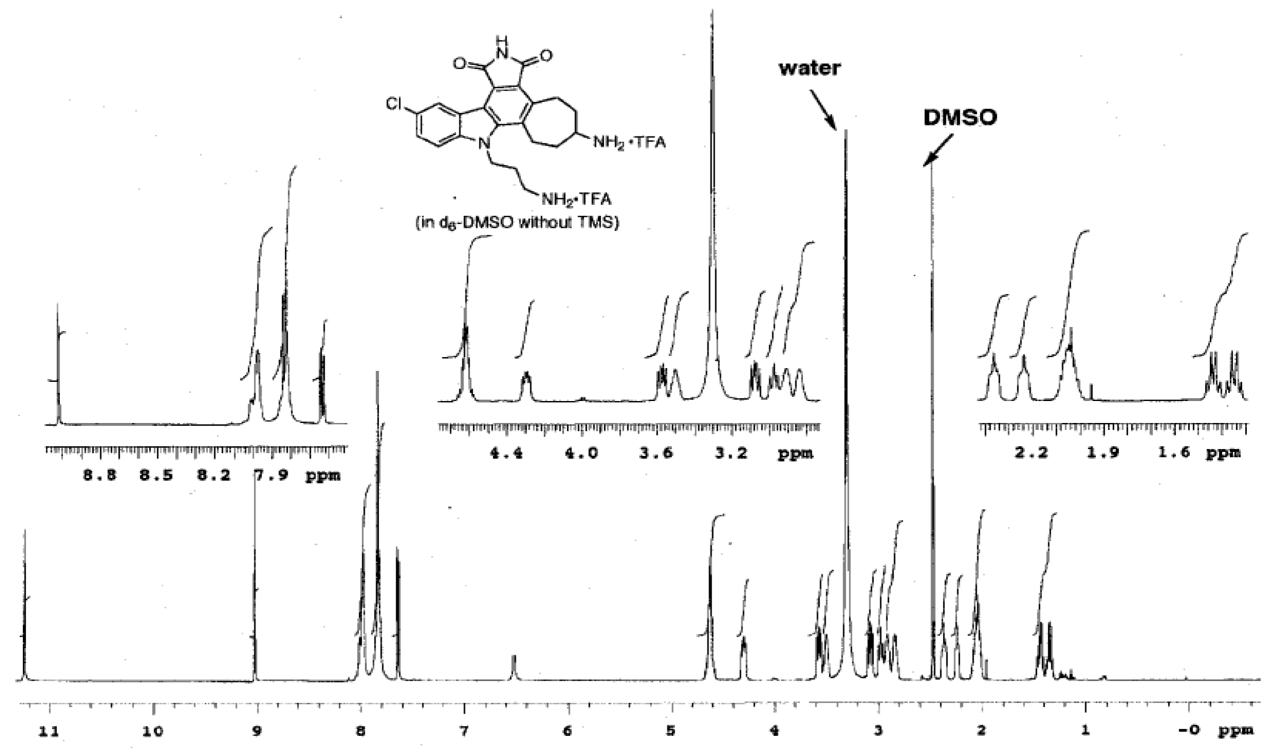

water
DMSO
(in d6-DMSO without TMS)
NH2•TFA
NH2•TFA
Cl
8.8 8.5 8.2 7.9 ppm
4.4 4.0 3.6 3.2 ppm
2.2 1.9 1.6 ppm
11 10 9 8 7 6 5 4 3 2 1 -0 ppm

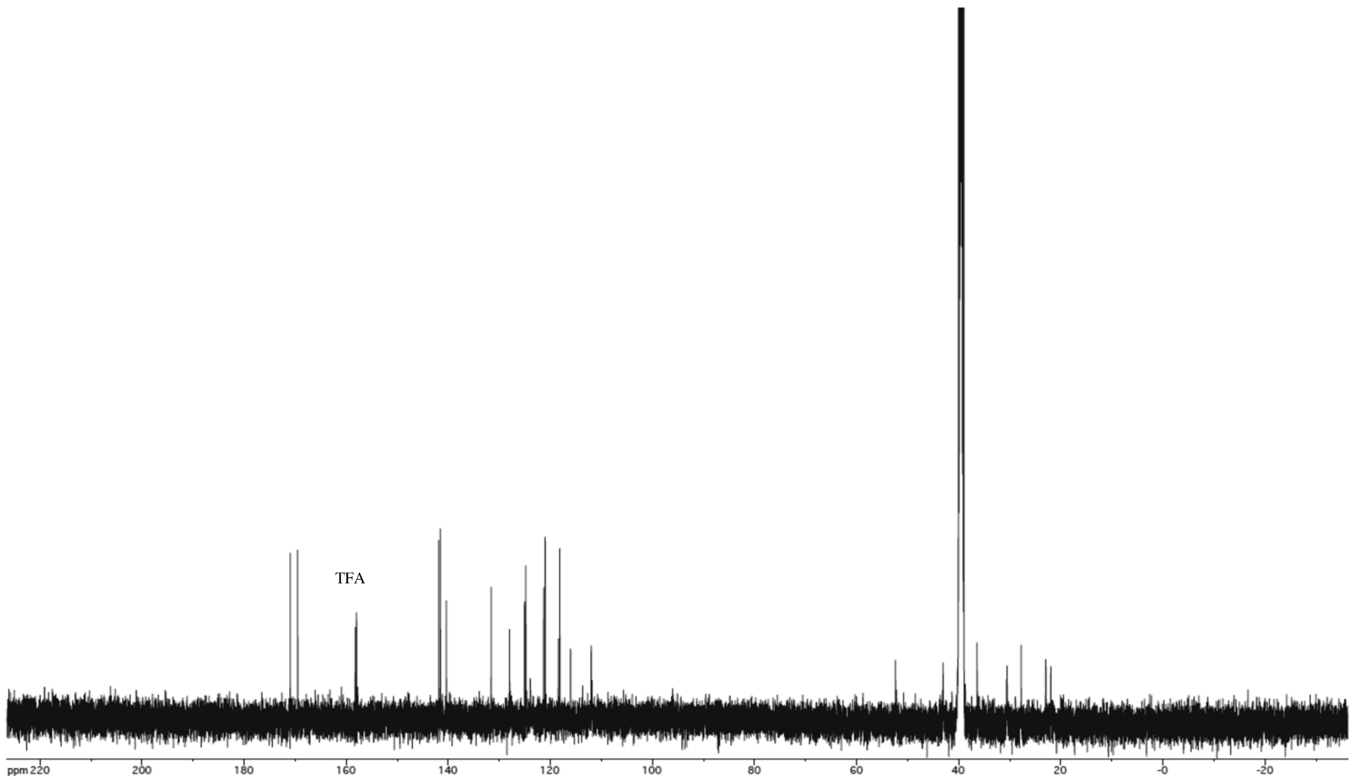

TFA
ppm 220 200 180 160 140 120 100 80 60 40 20 0 -20

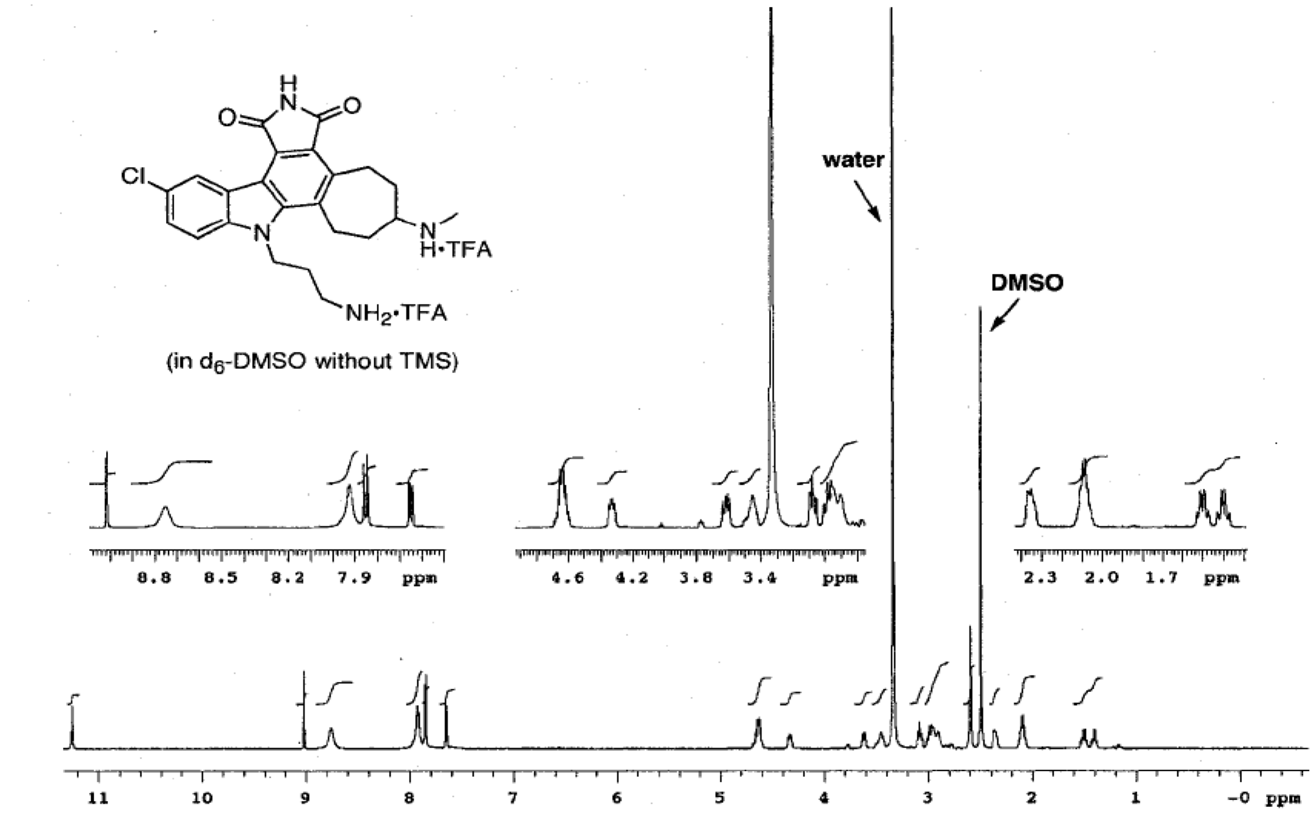
Cl
H•TFA
NH2•TFA
(in d6-DMSO without TMS)
water
DMSO
ppm

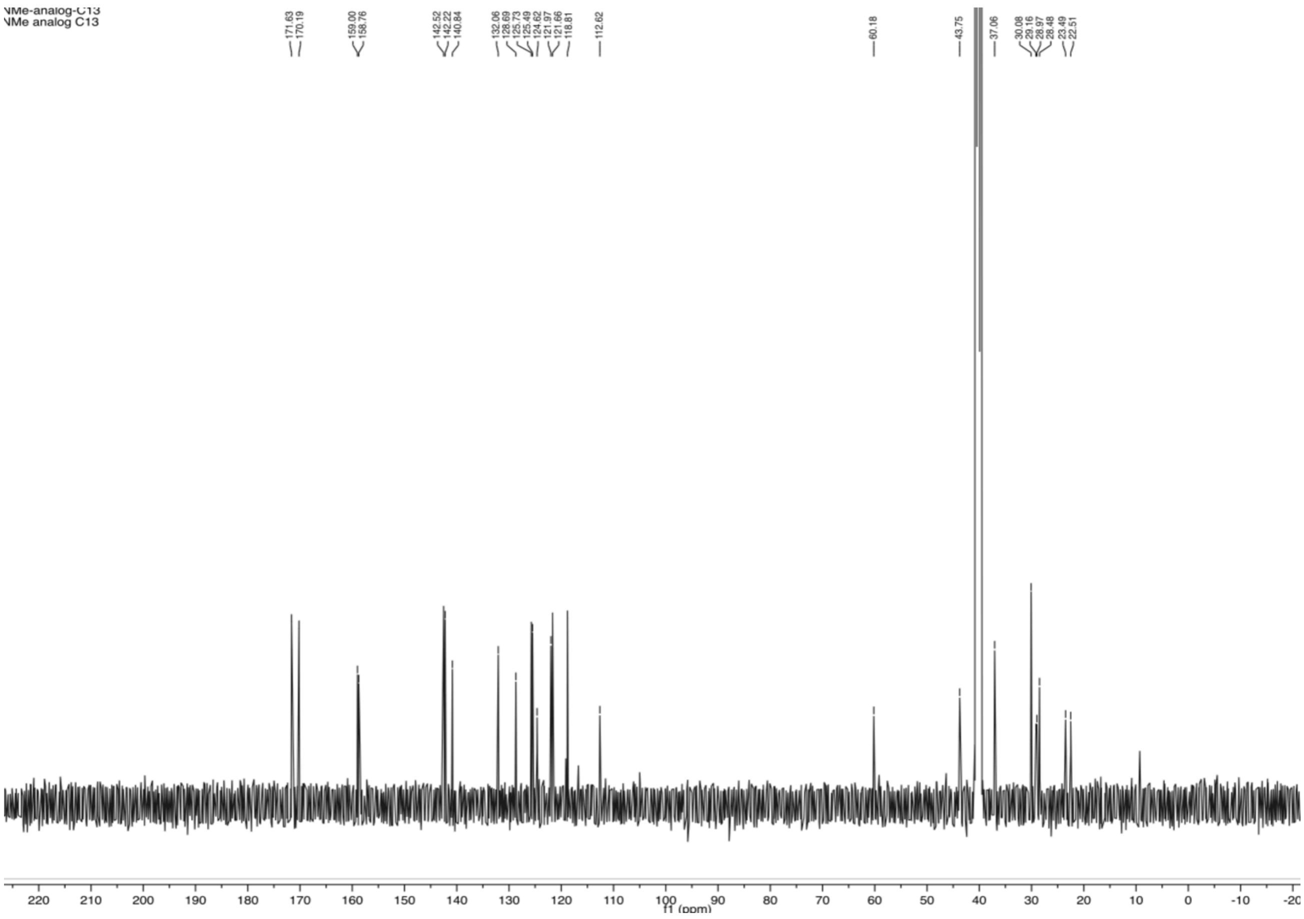
NMe-analog-C13
NMe analog C13
171.63
170.19
159.00
158.76
142.52
142.22
140.84
132.06
128.69
125.73
125.49
124.62
121.97
121.66
118.81
112.62
60.18
43.75
37.06
30.08
29.16
28.97
28.48
23.49
22.51
f1 (ppm)

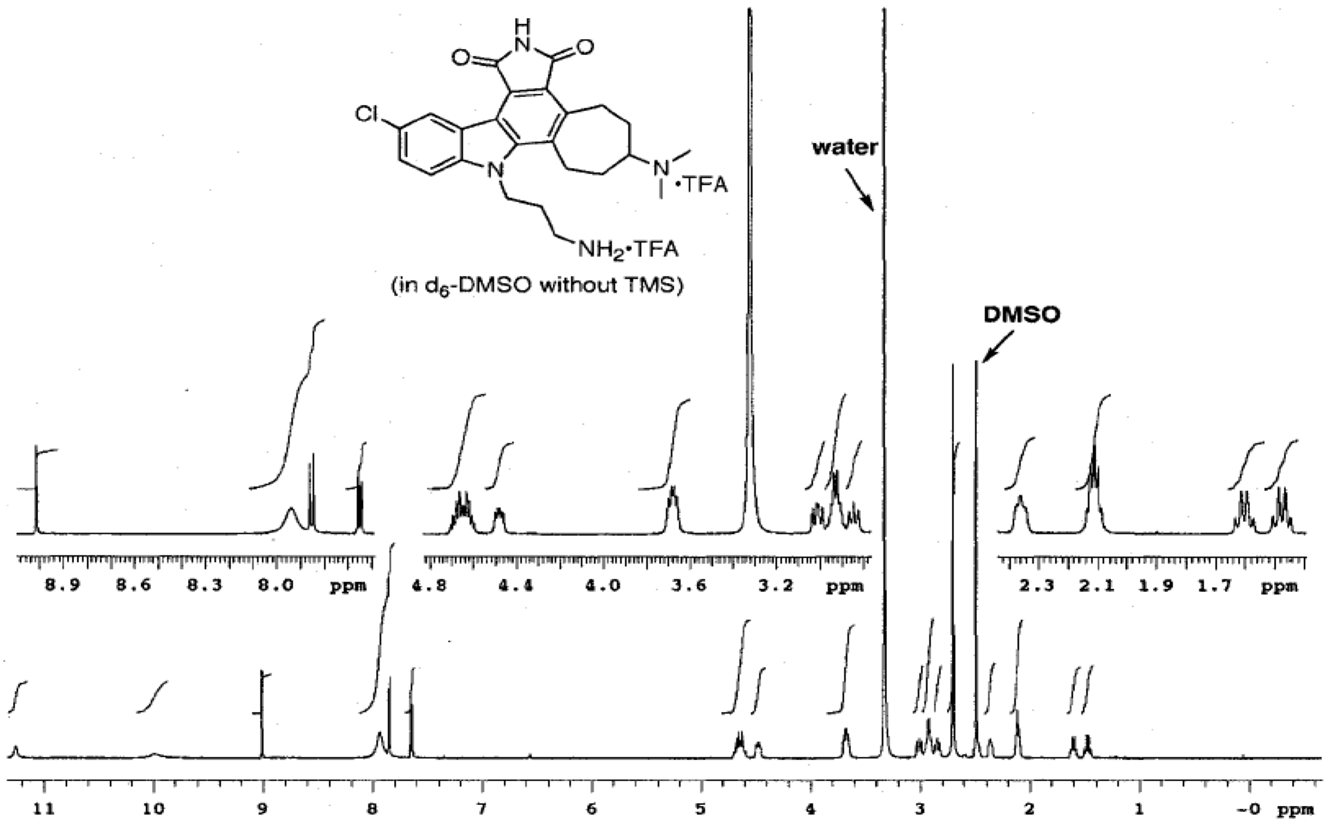
Cl
NH₂•TFA
•TFA
(in d6-DMSO without TMS)
water
DMSO
ppm

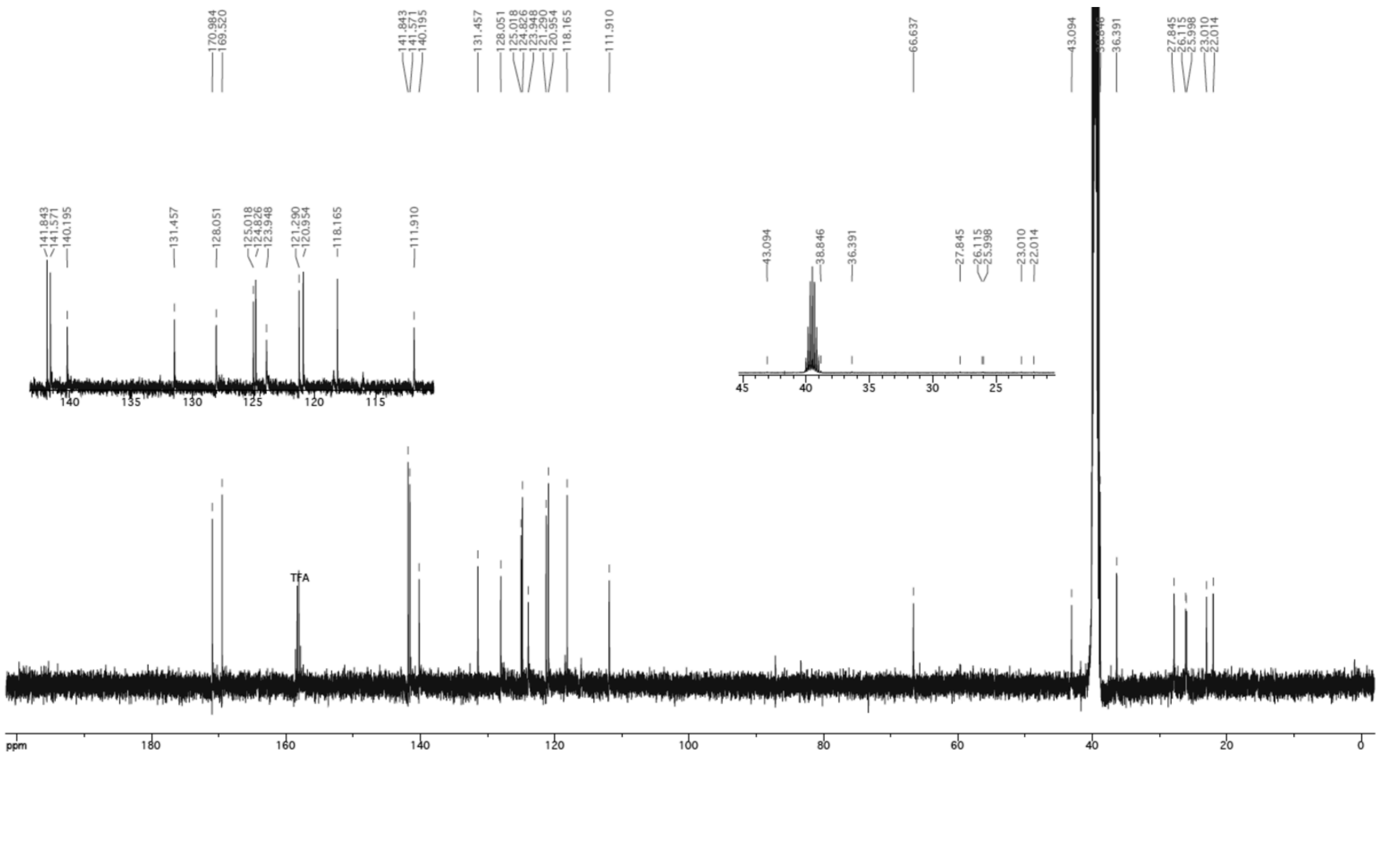
170.984
169.520
141.843
141.571
140.195
131.457
128.051
125.018
124.826
123.948
121.290
120.954
118.165
111.910
66.637
43.094
38.846
36.391
27.845
26.115
25.998
23.010
22.014
TFA
ppm

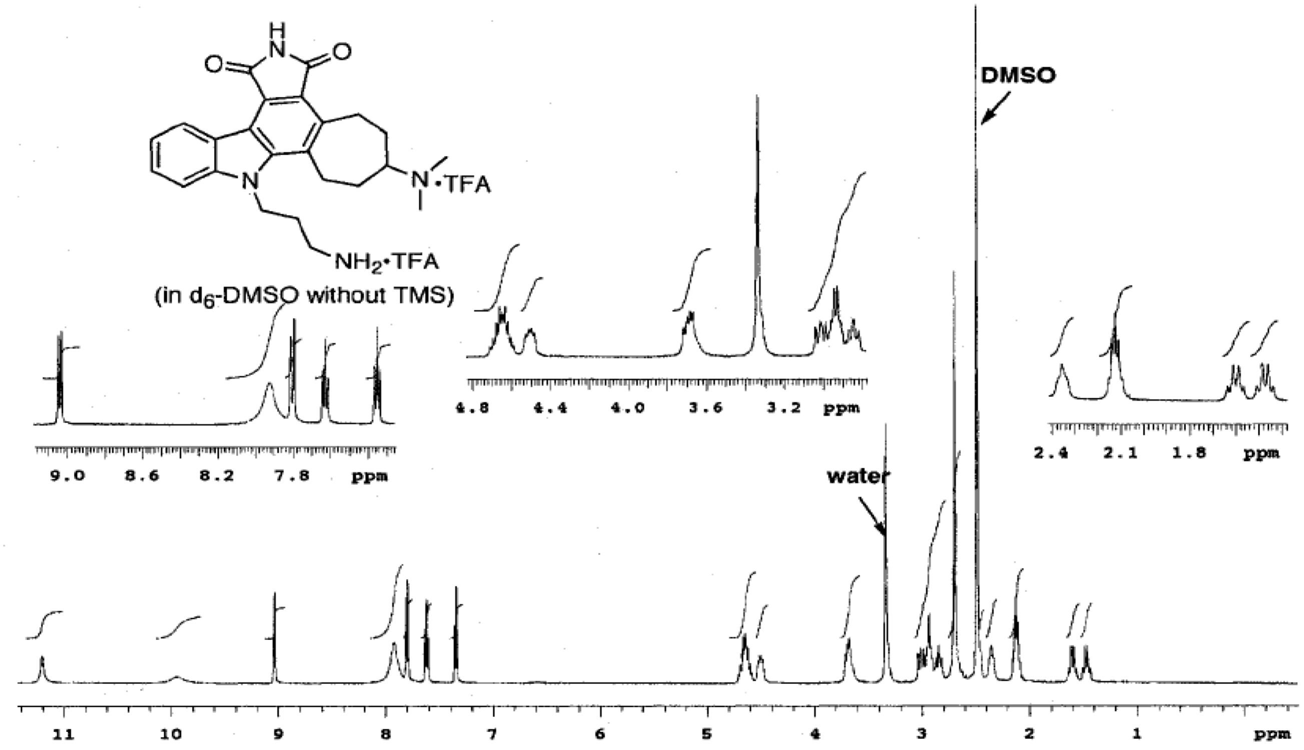
NH2•TFA
N•TFA
(in d6-DMSO without TMS)
DMSO
water
ppm

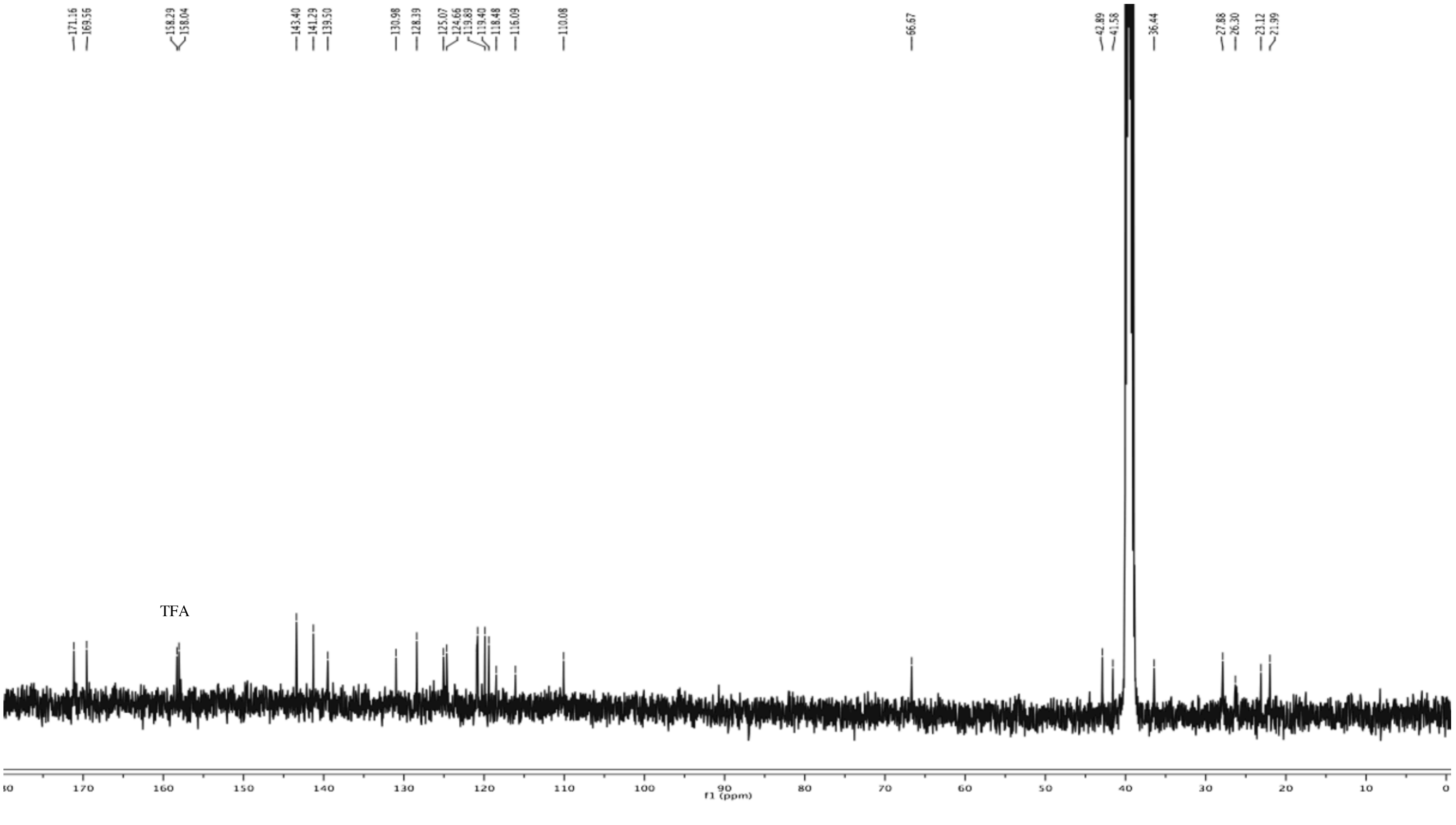
171.16
169.56
158.29
158.04
143.40
141.29
139.50
130.98
128.39
125.07
124.66
119.89
119.40
118.48
116.09
110.08
66.67
42.89
41.58
36.44
27.88
26.30
23.12
21.99
TFA
f1 (ppm)

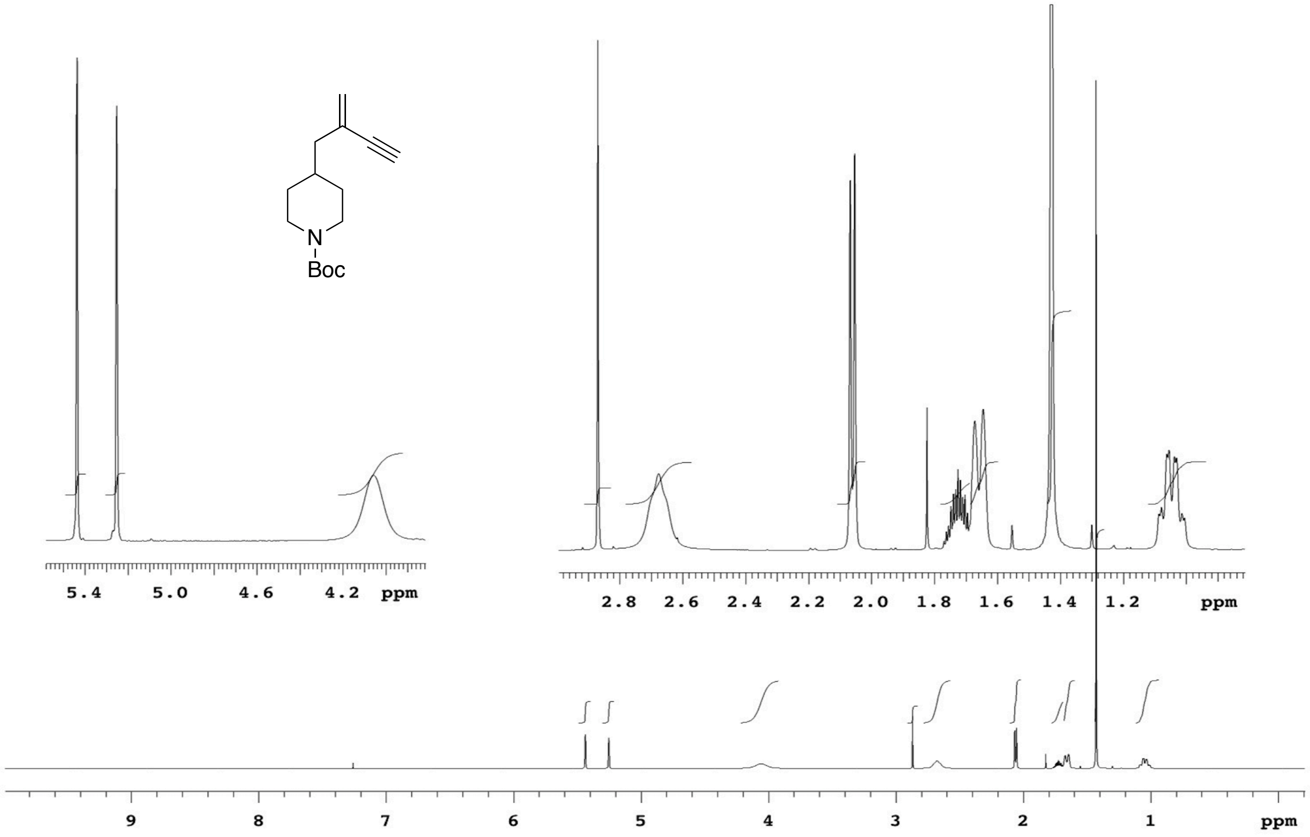
N
Boc
5.4 5.0 4.6 4.2 ppm
2.8 2.6 2.4 2.2 2.0 1.8 1.6 1.4 1.2 ppm
9 8 7 6 5 4 3 2 1 ppm

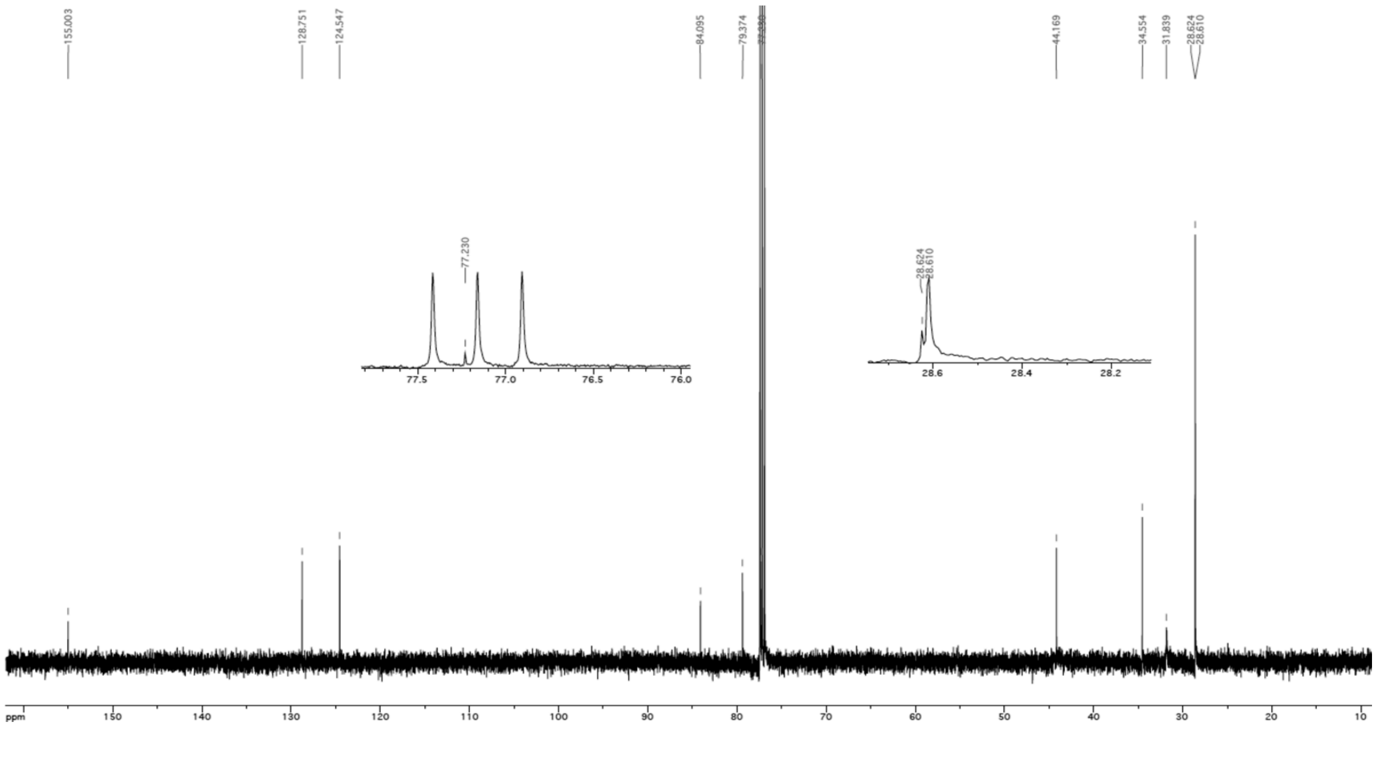
155.003
128.751
124.547
84.095
79.374
44.169
34.554
31.839
28.624
28.610
77.230
77.5 77.0 76.5 76.0
28.624
28.610
28.6 28.4 28.2
ppm 150 140 130 120 110 100 90 80 70 60 50 40 30 20 10

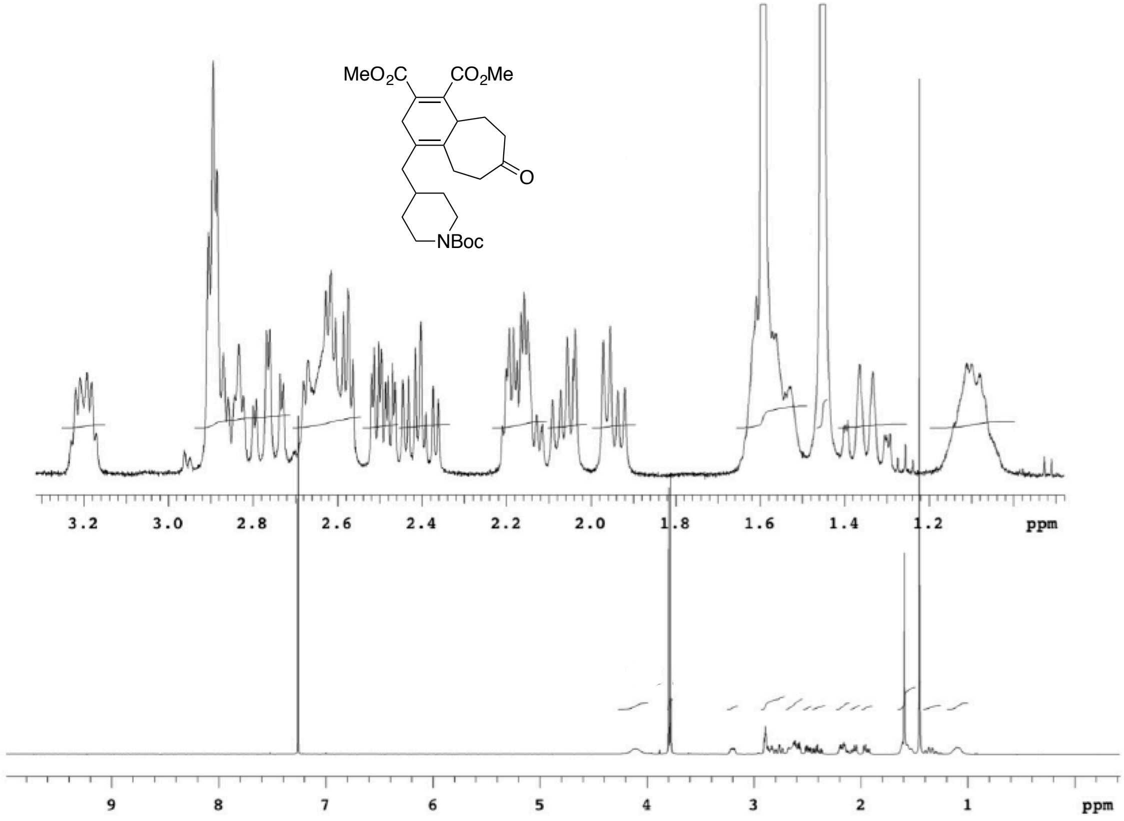
MeO2C
CO2Me
O
NBoc
ppm
ppm

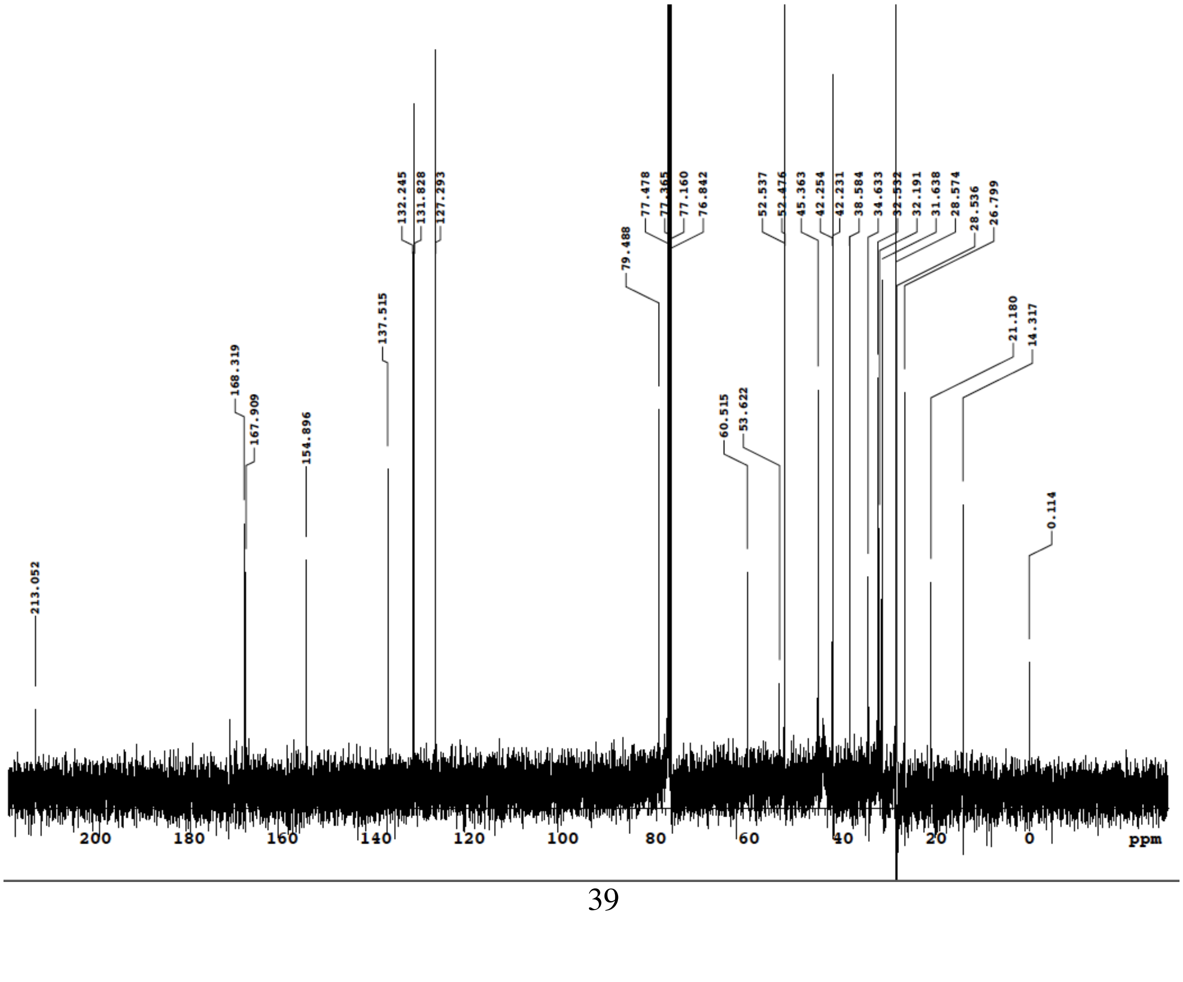
213.052
168.319
167.909
154.896
137.515
132.245
131.828
127.293
79.488
77.478
77.365
77.160
76.842
60.515
53.622
52.537
52.476
45.363
42.254
42.231
38.584
34.633
32.532
32.191
31.638
28.574
28.536
26.799
21.180
14.317
0.114
ppm

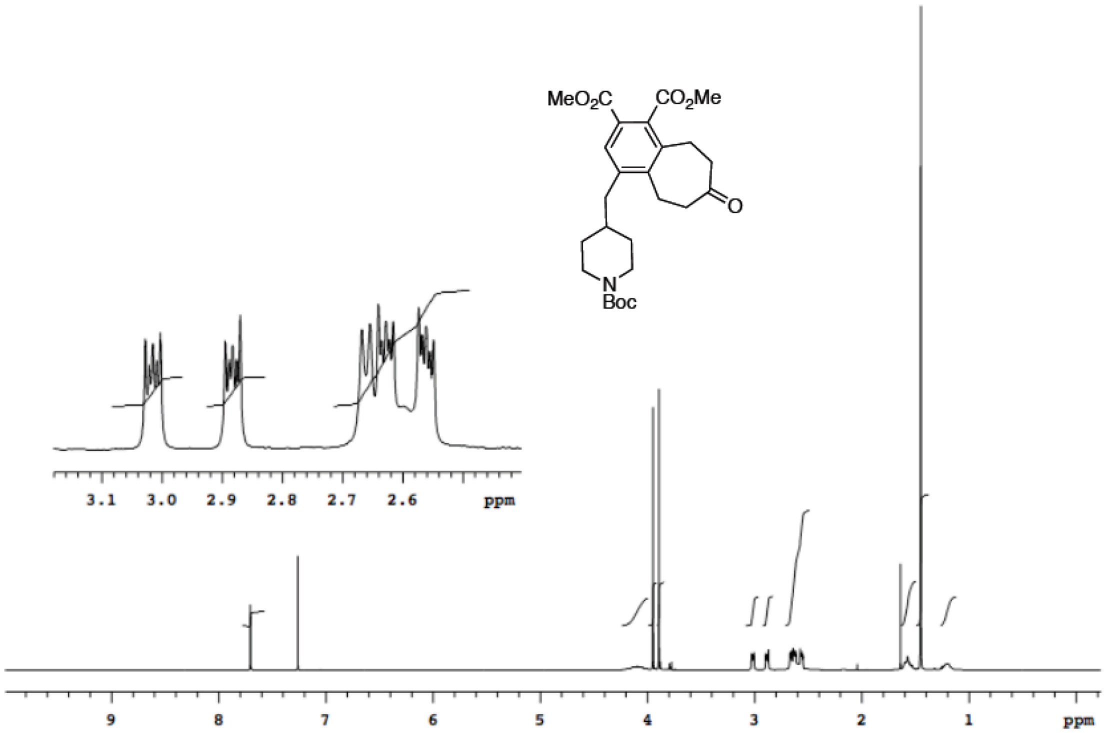
MeO2C
CO2Me
O
N
Boc
3.1 3.0 2.9 2.8 2.7 2.6 ppm
9 8 7 6 5 4 3 2 1 ppm

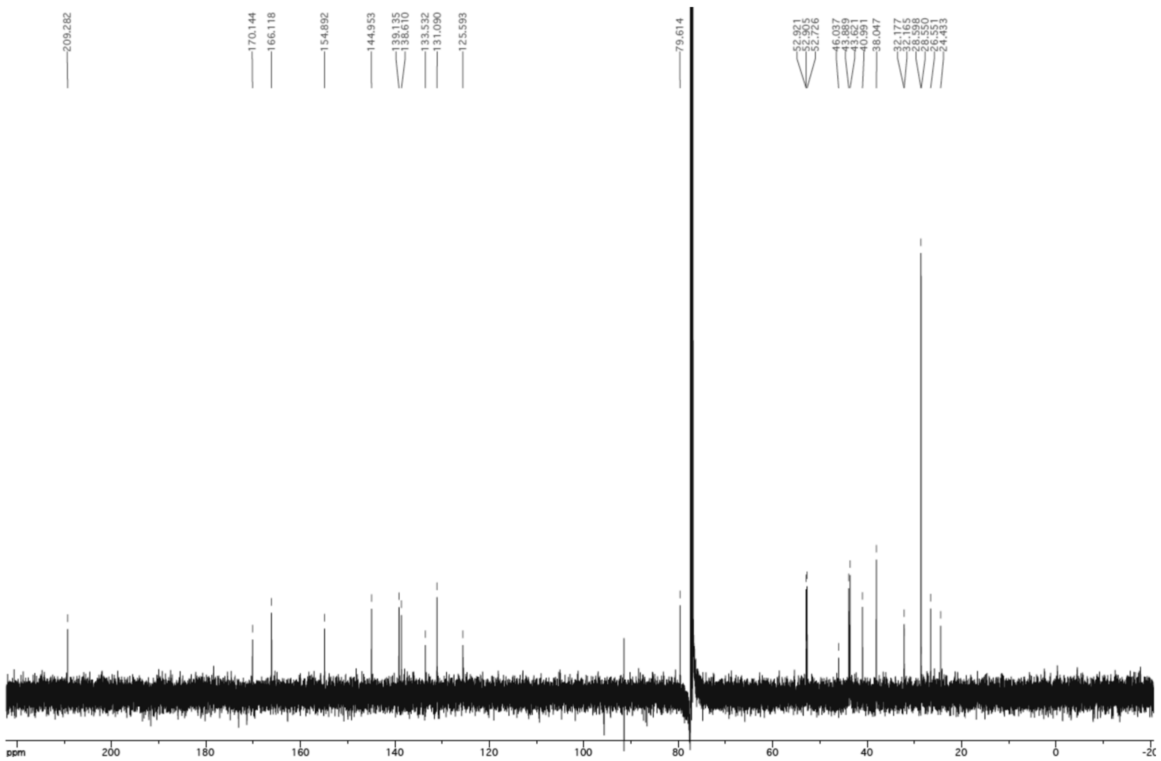
209.282
170.144
166.118
154.892
144.953
139.135
138.610
133.532
131.090
125.593
79.614
52.921
52.905
52.726
46.037
43.889
43.621
40.991
38.047
32.177
32.165
28.598
28.550
26.551
24.433
ppm 200 180 160 140 120 100 80 60 40 20 0

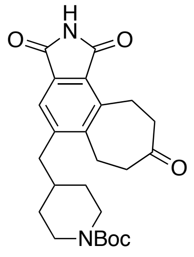

O
H
N
O
O
NBoc

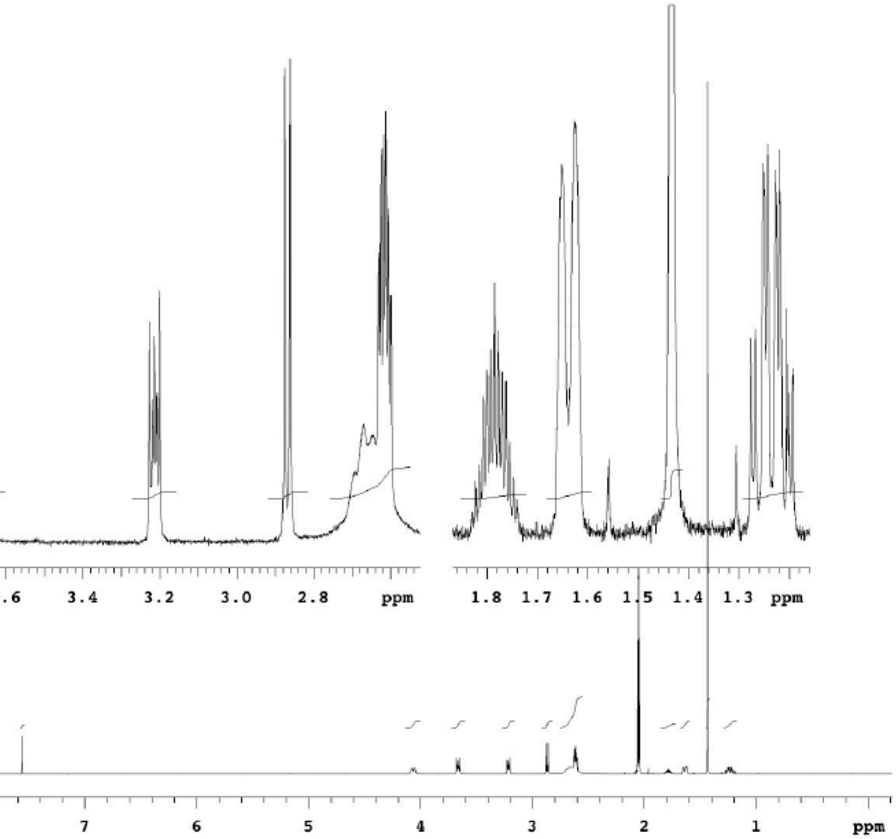

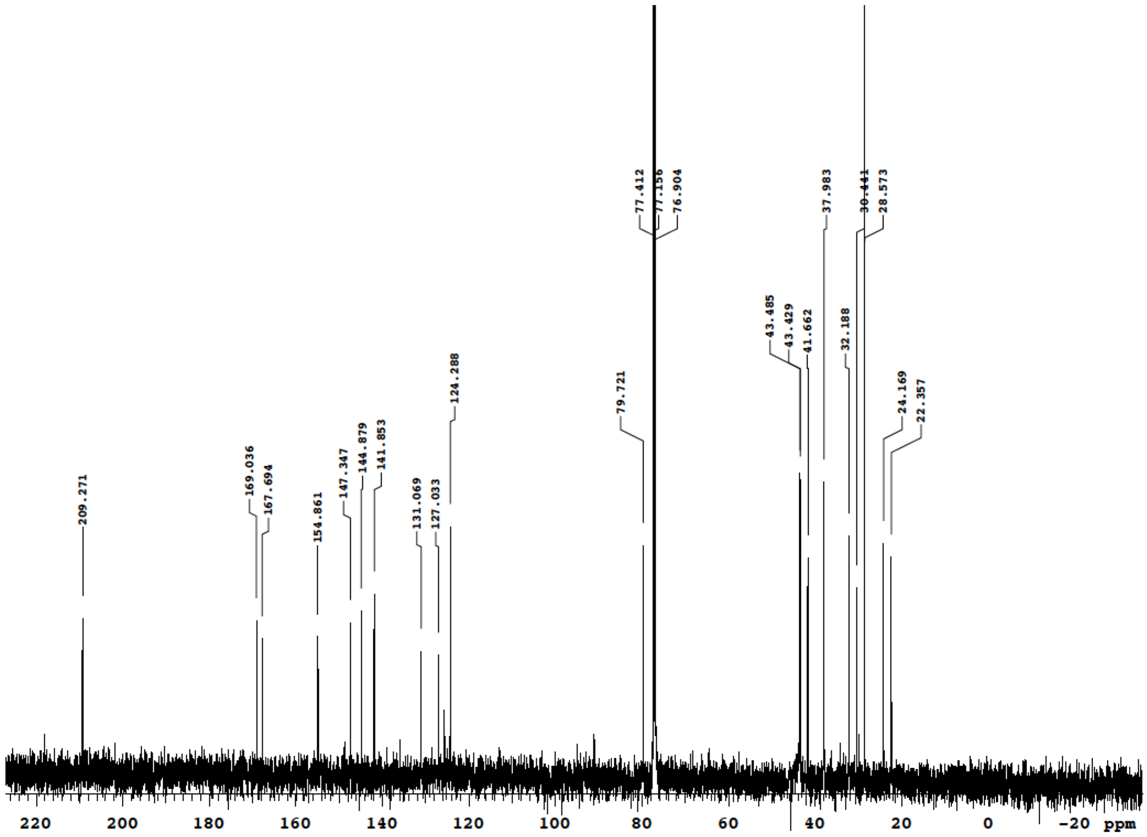

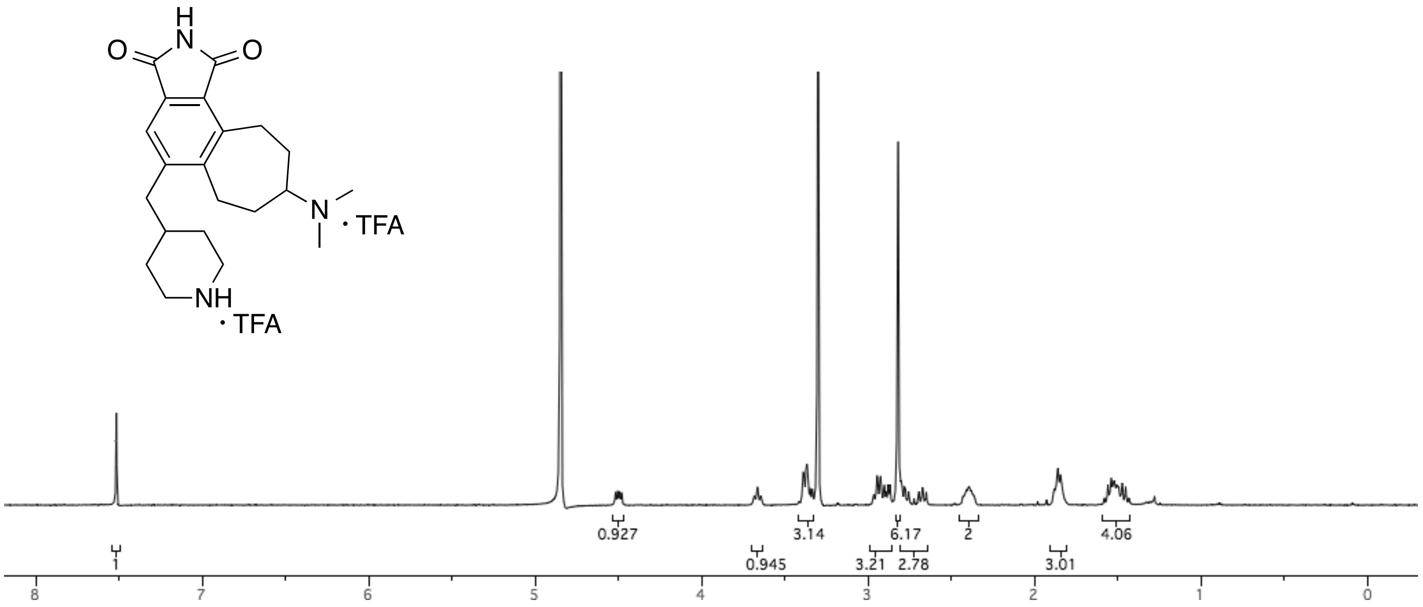
H
N
O
O
N
· TFA
NH
· TFA
1
0.927
0.945
3.14
3.21
6.17
2.78
2
3.01
4.06
8 7 6 5 4 3 2 1 0

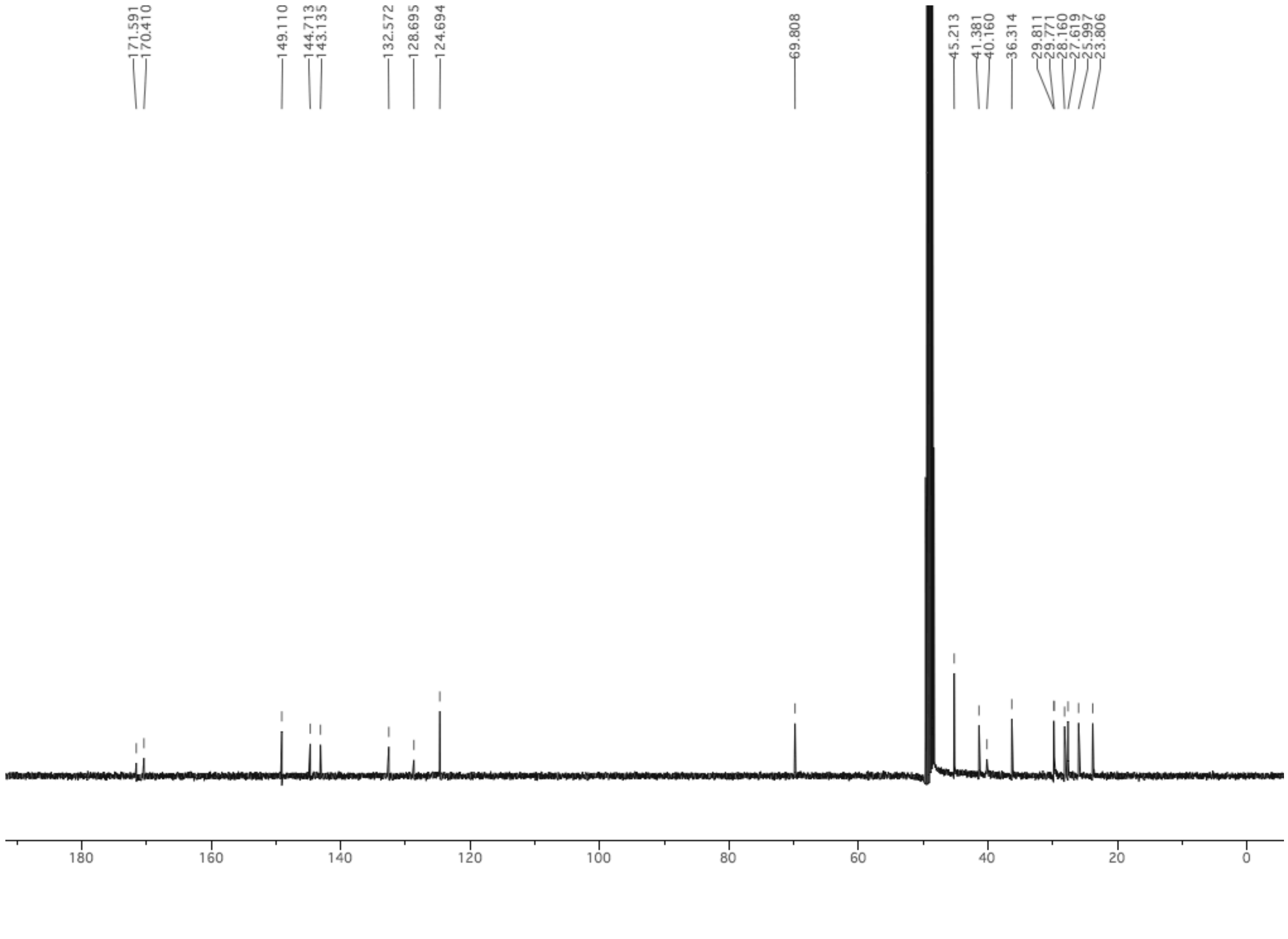
171.591
170.410
149.110
144.713
143.135
132.572
128.695
124.694
69.808
45.213
41.381
40.160
36.314
29.811
29.771
28.160
27.619
25.997
23.806
180 160 140 120 100 80 60 40 20 0
